# Beyond the Economic Gaze: Childbearing During and After Recessions in the Nordic Countries

**DOI:** 10.1007/s10680-020-09570-0

**Published:** 2020-11-19

**Authors:** C. L. Comolli, G. Neyer, G. Andersson, L. Dommermuth, P. Fallesen, M. Jalovaara, A. Klængur Jónsson, M. Kolk, T. Lappegård

**Affiliations:** 1 University of Lausanne, Quartier UNIL-Mouline, Bâtiment Géopolis, Bureau : 5321, CH-1015 Lausanne, Switzerland; 2 Stockholm University, Stockholm, Sweden; 3 University of Oslo, Oslo, Norway; 4 ROCKWOOL Foundation, Copenhagen, Denmark; 5 University of Turku, Turku, Finland; 6 The Research Department at Statistics Norway (SSB), PO Box 2633, St. Hanshaugen, 0131 Oslo, Norway

**Keywords:** Fertility, Childbearing, Recession, Economic uncertainty, Welfare uncertainty, Nordic countries

## Abstract

During the 2010s, fertility rates fell across the Nordic region. The onset of these declines seems linked to the Great Recession of 2008–2009, but their continuation cannot easily be linked to subsequent economic change. The 1990s, too, brought episodes of economic crises to the Nordic region that were followed by different degrees of fertility decline. In this study, we provide an empirical overview of parity-, age- and education-specific fertility developments in the five Nordic countries in the wake of the economic recessions in 2008 and the early 1990s, respectively. We demonstrate a high degree of heterogeneity in fertility developments across countries after 1990, whereas after 2008, the trends are much more similar across the five countries. Likewise, the educational differences in birth hazards that characterized the developments after 1990 were much smaller in the initial years after 2008–2009. This reversal from heterogeneity to homogeneity in the fertility response to recessions calls for an expansion of theories on the cyclicality of fertility in relation to uncertainty and economic and social change. In our discussion, we consider the role of a set of factors that also incorporates the state, crisis management, and perceptions of economic and welfare uncertainty.

## Introduction

The Nordic countries—Denmark, Finland, Iceland, Norway, and Sweden—are widely recognized for having generous family policies, protective and employment-oriented welfare systems, high rates of labor-force participation among both men and women, and, until very recently, comparatively high fertility levels. After the large decline in fertility across Western countries in the 1970s, total fertility rates (TFRs) in the Nordic countries remained in the range between 1.7 and 2.3 children per woman for several decades (1980–2010). Only in Denmark and Sweden did it temporarily fall to 1.4 and 1.5 children per woman in the mid-1980s (Denmark) and mid-1990s (Sweden), respectively. During the same period, most other countries in Europe consistently registered fertility rates below 1.7, and many Southern and Eastern European countries even experienced lowest-low fertility rates below 1.3 children per woman (Kohler et al. 2002). Scholars have tended to attribute the high fertility in the Nordic countries to their comprehensive welfare states and their family policies that support the reconciliation of employment and family commitments for both women and men (McDonald 2000; Esping-Andersen et al. 2002). It has been argued that these policies enabled the Nordic countries to maintain their period fertility around the replacement level. However, this picture now seems to be set to change. Since 2010, the TFR has been declining in all Nordic countries, hitting the historically low levels of 1.35 children per woman in Finland in 2019, 1.53 in Norway in 2019, and 1.71 in Iceland in 2017 (Nordic Statistical Central Bureaus 2018, 2019 and 2020). This ongoing decline in period fertility came largely unexpectedly, and it represents a conundrum that demographers have yet to address. The timing of the onset of declines suggests a link to the Great Recession (i.e., the financial and economic crisis that hit Europe after 2007). To a large extent, the Nordic countries’ have experienced relative financial solidity based on a combination of low public debts, efficient public administration, and a competitive business sector. At the same time, the countries are small, export-oriented economies, and vulnerable to external financial and demand shocks (Lin et al. 2014). After the onset of the crisis, a few crucial manufacturers went bankrupt (e.g., Saab in Sweden) or slipped into great financial difficulties (e.g., Nokia in Finland). Unemployment rates briefly tripled in Iceland, doubled in Denmark, and rose substantially in the other countries. Governments intervened with stimulus packages estimated to range between 0.7 percent and 2.7 percent of GDP in 2009–2010 (Lin et al. 2014).

Based on aggregate data, the cyclicality of fertility rates in relation to business cycles has been studied quite extensively in recent years. Empirical research generally shows that TFRs tend to decline during economic downturns in response to increasing unemployment and labor-market insecurity, dropping prices in the housing market, declines in consumer confidence, and increased financial uncertainty (Schneider 2015; Comolli 2017; Örsal and Goldstein 2018) but that extensive welfare-state support may alleviate much negative impacts of economic depressions on fertility (Myrdal 1945; Sobotka et al. 2011). In general, most studies find that fertility declines are temporary and constitute mainly a postponement of births among younger women (for a review see Sobotka et al. 2011). Several studies also indicate that fertility responses may vary across recession episodes, countries, parities, age groups, and social strata (Sobotka et al. 2011; Neels et al. 2013; Comolli 2018; Bellido and Marcén 2019). Economic downturns tend to more negatively affect first births among younger adults who have the opportunity to postpone childbearing until economic and labor-market security has been restored (Andersson 2000). Higher parities and births to older women are usually, but not always (see Comolli and Bernardi 2015 and Caltabiano et al. 2017) less influenced by business cycles (Sobotka et al. 2011; Goldstein et al. 2013; Comolli 2017). Economic recessions tend to have different impacts on the fertility of highly and low-educated women, but these differences may vary across countries (Sobotka et al. 2011; Neels et al. 2013).

The most common rationale for the decline of fertility during periods of economic turbulence comes from economics and concerns the financial costs of children and opportunity costs for women and families (Becker 1960; Ranjan 1999). Economic theories presume that childbearing entails immediate expenses and long-term financial and time commitments. Therefore, when financial and labor-market insecurities rise, incomes drop, and career prospects deteriorate, individuals tend to postpone major irreversible commitments such as having a child.

In what follows, we compare changes in childbearing intensities in the Nordic countries. The recent Great Recession affected the Nordic countries to different extents, and, before that, they were differentially shaken by another common wave of economic turmoil in the early 1990s. The societal, welfare, and macroeconomic features shared by the Nordic countries over the past four decades allow us to apply the most-similar-case comparison (Przeworski and Teune 1970; Neyer and Andersson 2008) in two ways: (a) to investigate the development of birth rates across two economic crises and (b) across five similar welfare states.

We present a systematic comparison of long-term developments in birth rates during and after recessions in Denmark, Finland, Iceland, Norway, and Sweden, contrasting the childbearing response to two episodes of economic downturn: the 1990s crisis and the Great Recession of 2008. This has not been done previously within a systematic comparative framework across countries and time. Our first and principal aim is to provide an empirical description of fertility developments during and after these two recent episodes of economic crises in the Nordic region. Viewing our systematic depiction of childbearing risks in the context of the timing, strength, and length of fertility change together with the crisis management in which they were embedded offers an opportunity to reflect on the theoretical advancement needed to understand fertility developments under different economic conditions. Our second aim is to provide such a discussion.

We use harmonized longitudinal administrative register data to analyze the fertility histories of women in the five Nordic countries and examine their childbearing risk by age, parity, and education. We extend previous studies by including higher-order births, performing separate analyses for women aged 16–29 and 30–45, and distinguishing between different educational levels. Regarding the methods, we follow the previously tested approach (Hoem 1991, 1993; Andersson 1999, 2002, 2004; Kravdal 2002; Vikat 2004; Andersson and Kolk 2016; Jónsson 2017) of using event-history techniques to present parity-specific indices of childbearing risks over calendar years relative to a baseline year. By reconstructing population-level year-to-year changes in childbearing risks based on individual-level data, instead of relying on aggregate synthetic measures of fertility, we are better able to provide an in-depth contribution with the capacity to trace the various temporal and cross-country fertility dynamics that have been at play. An empirical test of theoretical hypotheses about the specific effects of macroeconomic conditions, austerity, or stimulus packages on fertility developments goes beyond the scope of this contribution. However, as a background to interpret the fertility developments under crises and assess theories of fertility behavior under such conditions, we first provide a country-specific overview of the economic crises and the measures that the countries took to tackle the crisis of the 1990s and the Great Recession. Second, we review previous research, sketch the existing theories, and propose additional mechanisms that broadens the view on the economy-childbearing nexus. This is followed by an in-depth presentation of the fertility trends themselves. In light of the apparent contradictions between traditional theoretical assumptions, narratives of the macroeconomic outlook during and after the recessions, and the most recent childbearing developments in the Nordic countries, we conclude with a discussion that expands the explanatory framework commonly used in studies of economic crises and sketches new dimensions for future research.

## Background

### The Two Recessions in the Nordic Countries

**Fig. 1:**
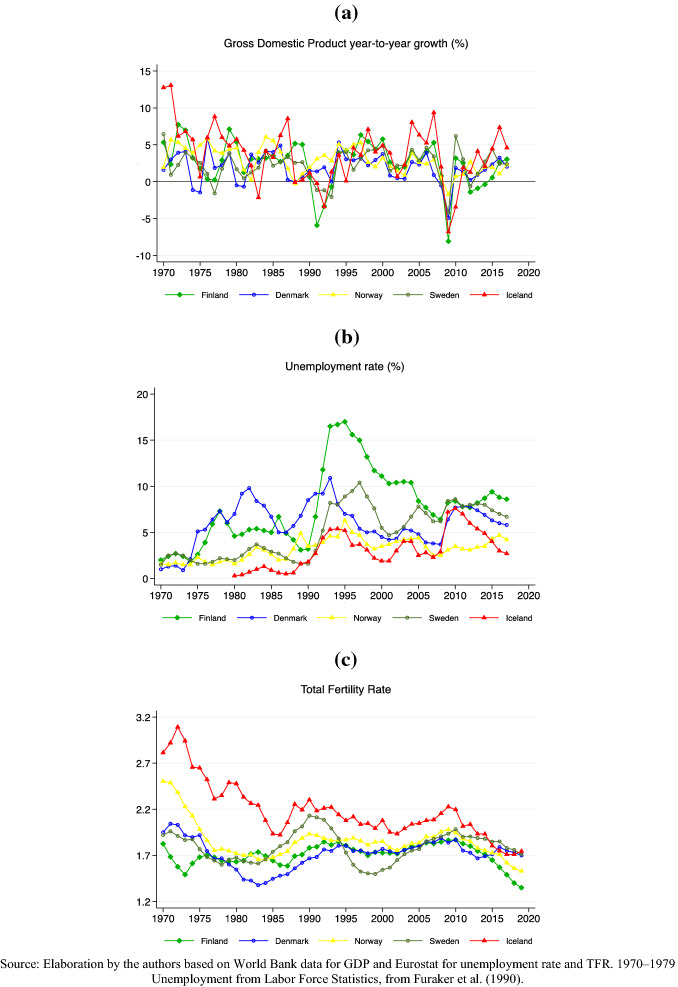
Annual GDP per-capita growth rate **(a)**, unemployment rate **(b)**, and total fertility rate **(c)** in the Nordic countries, 1970–2017 (2019)

Until the 1980s, the macroeconomic outlooks for the Nordic countries were remarkably similar in terms of public finance, economic growth, and employment rates (Andersen 1997). All five countries, for instance, had been able to avoid the mass-unemployment crises that plagued most European countries in the 1970s. Denmark exceptionally experienced unemployment rates above 5 percent in the mid-1980s, but it was not before the onset of the financial crisis of the early 1990s that the Nordic countries underwent a communal recession. To illustrate the development across countries and economic crises, we present the trends in the period 1970–2017 of two macroeconomic indicators that are commonly used to describe economic cycles—gross domestic product (GDP) growth rates and unemployment rates—together with total fertility rates in 1970–2019 (Fig. 1; “Appendix Table 2”). Despite the shared timing and the global roots of the financial crisis, the 1990s recession was heterogeneous across the five Nordic countries, partly due to national conditions at the onset of the recession and its subsequent management (Ólafsson et al. 2019). GDP growth (Fig. 1a) shows that during the early 1990s, the Nordic countries split into two groups: the ones that did not register negative growth of their GDP at all (Norway and Denmark) and the ones that experienced negative economic development and partly severe drops of their GDP growth rates between 1991 and 1993 (e.g., Finland in 1991: -5.9 percent; Iceland in 1992: -3.4 percent; Sweden in 1993: -2.1 percent). By contrast, all five countries experienced negative GDP growth in 2009. The negative development was considerably worse during the most recent crisis episode, with GDP growth rate falling by 8.3 percent in Finland; 6.9 percent in Iceland; 5.2 percent in Sweden; 4.9 percent in Denmark; and 1.6 percent in Norway—all in 2009. GDP growth rates were positive again in 2010, although another economic setback occurred in Sweden in 2012 and in Finland in 2012–2014. In Norway, too, the recovery in GDP annual growth has been more modest after the Great Recession compared to after the 1990s crisis. Figure 1b shows that negative peaks in production growth are mirrored by rapid increases in unemployment rates. During the 1990s recession, the unemployment rate doubled in three of the five Nordic countries. The steepest rise in unemployment was registered in Finland, where the rate grew from 3.2 percent in 1990 to 16.7 percent in 1994, followed by Sweden, with an increase from 1.6 percent in 1990 to 8.2 percent in 1993. Except for Denmark and Norway, the unemployment rates of the Nordic countries have never returned to their low rates of the early 1980s. During the Great Recession, unemployment did not rise as dramatically as two decades earlier, but the recovery also seems slower, and in all five countries, unemployment rates were higher in 2015 than in 2008. In Norway and Finland, unemployment rates even increased during 2012–2015.

Figure 1c shows that the fertility developments in the 1990s also differed across the five countries. Sweden had a more volatile TFR over the extended time period compared to the other countries (Hoem and Hoem 1996; Andersson 2004), while Finland displayed no fertility reaction to the 1990s recession (Andersson 2000; Vikat 2004; Neyer et al. 2006). Despite the weakness of the 1990s recession in Norway, the positive trend in fertility rates of the late eighties came to a halt after 1991. The fertility trends during the 1990s in Denmark are very similar to the Norwegian ones. All countries show a similar development of the TFR since the turn of the twenty-first century and, despite the brevity of the Great Recession, a remarkable and very persistent decline over the past decade, with TFRs in Finland, Norway, and Iceland falling below the levels of the 1980s and to all-time lows in recent years.

### The 1990s Recession and Its Management in the Nordic Countries

Despite the common roots in the global financial deregulation of the late eighties and early nineties, the crisis of the 1990s in the Nordic countries was also self-inflicted and very national in its developments and management. On the one hand, Denmark and Norway dealt with the deregulation more gradually and used, respectively, social-policy instruments and oil revenues to counterbalance the recession. On the other hand, Finland and Sweden deregulated their financial markets more abruptly and later suffered from severe currency and banking crises (Jonung et al. 2009).

In Finland, the crisis was additionally aggravated by the collapse of the Soviet Union, which generated a sudden drop in the exports thereby directed. The Finnish unemployment rate increased dramatically, and in the first half of the 1990s, the perception of job insecurity among the employed rose rapidly (Nätti et al. 2001; Rasmussen et al. 2019). Although the Finnish welfare state overall retained its functions (Heikkilä and Uusitalo 1997), disposable income decreased as tax rates rose. Prior to the crisis, Finland had introduced a home-care allowance (HCA) paid to parents whose children under the age of three were not in publicly provided day care and had also granted the rights for day-care places for all children under three by 1990. During HCA eligibility, employed parents have, vis-à-vis the employer, the right to take childcare leave and the security of returning to their job. As the crisis hit, the HCA was increased by 30 percent in order to restrict expenditure for public childcare, and parental leave for mothers and fathers was extended (Hiilamo and Kangas 2009; Haataja 2005). Like most social benefits, the HCA was cut (by 20 percent) in 1996, and eligibility requirements for it and for parental leave were tightened (Vikat 2004; Haataja 2005; Kangas 2019).

In Sweden, the banking sector was rapidly deregulated in the late 1980s, and, as a response, the amount of credit expanded quickly, and the economy overheated. In 1990, several financial institutions went bankrupt, followed by more bankruptcies in the following years. The crisis hit the economy broadly, spreading from the private to the public sectors (Palme 2019). Male and female employment dropped substantially and housing-property prices collapsed. The budgets for most publicly funded social services were subsequently cut and almost all cash transfers, including parental leave, were substantially reduced. This generated significant hardship for individuals and especially for households with children (Palme 2019). Despite later revisions of the cuts, heavy investments in active labor-market policies and in secondary and tertiary education, and an extension of rights to childcare services, employment levels did not get back to pre-crisis levels until the end of the 1990s.

The Danish recession hit earlier than the Swedish one. Through too-high private and public spending, a persistent savings deficit, and rapid wage growth throughout the early 1980s, the Danish economy overheated in 1987 (DØR 2007). To try to stem the overheating, Denmark introduced a series of reforms in 1986–1987 that capped access to credit and mortgages, increased taxation of financial institutions to discourage speculation, and encouraged saving through large national pension funds. The timing of the reforms likely contributed to increasing the economic slowdown (DØR 2007). From 1987 to 1993, Denmark saw seven lean years with little to no growth in GDP together with rising unemployment topping out in 1993 at around 12 percent of the labor force. From 1994, unemployment began to decrease. This coincided with a series of reforms of active labor-market programs, increased eligibility requirement for the unemployment insured, “flexicurity” policies that eased both hiring and firing, and the introduction of extensive paid parental leave, education, and sabbatical arrangements to stimulate job rotation (Madsen 2006; Compton and Madsen 2001). Further, the economy received a “kickstart” in 1994 through lowering the marginal tax rate and increasing the tax-deductible share of interest rates on loans (DØR 2007), which increased both GDP growth as well as national production. From 1994, Denmark saw steady growth and decreasing unemployment throughout the rest of the decade.

Similar to Denmark, the recession came earlier in Norway and was reinforced by an oil-price collapse in 1986. Housing prices decreased, and interest rates rose. In addition, the decline in demand for oil following the collapse of the Soviet Union affected Norway as well. In 1990, to mitigate volatility stemming from fluctuating oil prices, Norway established its Oil Fund. A percentage of the fund revenues was allocated to the state budget, giving Norway a unique scope of action. Efficiency in the bank sector increased, and tax reforms to stimulate private investment were implemented. Contrary to Sweden, where social services were cut and the public sector shrunk, Norway introduced a comprehensive employment strategy. It tightened rules on temporary employment, expanded employment in the public sector and in childcare, extended parental leave from twelve to forty-four weeks at 100 percent wage compensation (in 1994), and prolonged unemployment benefits from one to two years (in 1997; Dølvik and Oldervoll 2019). Additionally, it renewed its vocational-training system and expanded higher education further. These reforms stimulated employment and strengthened the welfare state, so that Norway emerged from the crisis with a welfare state and employment regime that was significantly more comprehensive and “Nordic” than the ones before the crisis (Dølvik and Oldervoll 2019).

Finally, with Iceland being a very small economy vulnerable to external shocks, the Icelandic business cycle is closely tied to the ups and downs in the global economy (Einarsson et al. 2015, 2016). Prior to the twin currency and bank crises in 1993, the Icelandic economic activity had been moving in parallel to size of fish catches and shocks to trade. The country had also experienced persistent inflation, depreciation of its currency, and high interest rates in the period leading up to the economic recession. Combined with a global economic downturn, the vulnerability of the Icelandic economy led to a relatively short-lived recession, but one that had significant impact on the labor market and, importantly, led to considerable emigration (Einarsson et al. 2015). In the upswing of the economy and the height of unemployment, social benefits, including child benefits, were reduced, and user fees for health and education increased. These restructurings were later partly countered by introducing and expanding gender-equal parental leave during the late 1990s (Eydal and Ólafsson 2008; Ólafsson 2003).

In sum, in line with the Nordic conception of a universalistic welfare state, all Nordic countries used active labor-market policies and investments in education to mitigate the consequences of the crisis of the early 1990s. They also partially expanded social services, such as childcare. Denmark, Norway, and Iceland extended their parental-leave policies. But there were also cuts and tightening of access to welfare support especially in Sweden and Finland. Despite these commonalities, the degree of investment and of retrenchment varied substantially across the countries and across the policies involved. However, the social-policy steps taken during and in the aftermath of the crisis made the welfare states of these countries more similar than they were before the crisis.

### The 2000s Great Recession and Its Management in the Nordic Countries

The Great Recession of 2008 was a global crisis. Its roots are to be found in the subprime crisis that originated in the USA in the summer of 2007. It rapidly spread to the other side of the Atlantic through contagion in the banking system. After the European financial markets nearly collapsed in the fall of 2008, the crisis reached the real economy through the abrupt reduction in credit lending by banks to firms. This translated into negative economic growth and dramatically rising unemployment rates all around Europe. The five Nordic countries did not escape the recession, but, differently from the 1990s, they were spared the major drama that hit other parts of the continent. Moreover, the negative growth registered in 2009 originated in the global recession, and, as such, the crisis background has been more similar in the Nordic countries and less “national” compared to the 1990s crisis.

Iceland represents the exception, being one of the most-affected economies in the world (around 90 percent of the country’s financial system collapsed). In the years leading up to the crisis, the country saw an increase in private and public indebtedness, with an accumulated foreign debt around eightfold Iceland’s GDP. Housing prices had increased significantly; the trade deficit was large and the currency overvalued. In late 2008, consumption fell by roughly 20 percent, and in 2010, the unemployment rate rose to 7.6 percent (Ólafsson 2011; Einarsson et al. 2015). Social and political unrest followed, and Iceland had to undergo a comprehensive economic program that included cuts in social spending, including the parental-leave program. Despite welfare policies implemented during and after the crisis—such as debt relief and active labor-market programs or increases in child benefits—young families with children were among the hardest hit by the crisis. Emigration, as during the 1993 crisis, increased considerably and contributed to lowering unemployment (Ólafsson 2019).

The second-most-affected country in the group was Finland. First, when the Great Recession hit, the country had not fully recovered from the previous crisis. Second, idiosyncratic shocks, such as crises in the information- and communication-technology sectors (with Nokia), and the Western embargo on and recession in Russia, one of Finland’s largest trade partners, negatively affected the Finnish economy. Third, a rigid salary structure and rapid decline in competitiveness of Finnish workers relative to other countries made it more vulnerable (Suni and Vihriälä 2016). Finally, since Finland had entered the Eurozone in 2000, it could not devalue its currency as in the early 1990s and as the other Nordic countries did. Although the Great Recession was milder compared to the 1990s crisis, the subsequent recovery was less impressive, too. The employment situation hardly improved at all. Austerity measures implemented since 2011, including reduced services and raised fees for families, cut deeply into welfare provisions and partly “neo-familialized” parental responsibilities (Kangas 2019).

In Denmark, the 2008 recession led to large decreases in private spending and investments (Jensen and Johannesen 2017) as well as soaring unemployment rates. Beyond the general slowdown of the international economy, the crisis in Denmark was further magnified by the burst of a national housing-market bubble and the collapse of several large banks (Erhvervs- og Vækstministeriet 2013; Jensen and Johannesen 2017). As of 2013, it was estimated that the crisis had resulted in a bill on Danish society at the equivalent of 12 percent of one year’s GDP (Erhvervs- og Vækstministeriet 2013). GDP per capita only reached its pre-crisis level in 2016, and evidence indicates that younger people in particular suffered from long-term lower employment (Andersen et al. 2017; Jonassen 2019). Prematurely thinking being out of the recession in 2010, Denmark halted a stimulus package and enacted structural reforms that had been prepared before the crisis, aimed to strengthen and increase labor supply (Andersen 2019). This was done through cutting social benefits and tightening eligibility criteria. It affected individuals and households with low income and low education more than others (Andersen 2019).

The Great Recession in Sweden was mild compared to most other countries. However, large Swedish export-oriented companies had to reduce employment due to declining global demand, making unemployment rise. Despite the Swedish economy maintaining an overall good shape, by 2015 the unemployment rate had not returned to its pre-crisis levels. Unlike in the 1990s, the Swedish financial and construction sectors were not hit very hard, and the crisis did not spread to the public sector. The policy responses regarding the financial sector were similar to those of the 1990s but different regarding social policies (Palme 2019). Contrary to the 1990s crisis, there was no substantial retrenchment. In fact, the introduction of earned-income-tax deductions just prior to the Great Recession lowered the fiscal pressure on those in employment. As a consequence, and despite some cutbacks of social-transfer programs, financial hardship on the population remained modest and affected mostly the low educated and those outside employment (Palme 2019).

Finally, the slowdown in economic growth and the rise in unemployment rates during the Great Recession in Norway have been minor and mostly linked to the decline in oil prices. Using the revenues from the Oil Fund, the Norwegian government implemented several measures that buffered the increase in unemployment and greatly weakened the overall impact of the crisis on the population. Fiscal-stimulus packages, selective but sizable tax reliefs, increases in spending, and expansionary monetary policy reduced credit constraints from banks to families, relieved taxes, and stimulated investments in infrastructures (Dølvik and Oldervoll 2019). Despite those stimuli, eight years after the Great Recession, GDP growth rates in Norway remain modest and unemployment rates even increased again after 2012.

To sum up, compared to the crisis in the 1990s, the Great Recession came as an external shock that hit the Nordic countries at the same time and in a relatively similar fashion, apart from Iceland which was hit harder. The five countries reacted more quickly to the Great Recession and used their own experiences and those of other Nordic countries with previous policy interventions to deal with the recession. Overall, the crisis management of 2008–2009 deviated less across the countries than the one in the 1990s. The policy measures adopted after 2010 nevertheless varied. While Finland adopted austerity measures and welfare cuts after 2011, Sweden and Norway did not implement any welfare retrenchment. However, just prior to the crisis Sweden had strengthened those in employment and trimmed down benefit levels of those outside employment. Denmark introduced structural reforms after 2010 that produced similarly marked inequalities.

### The Business Cycle–Fertility Nexus: Theoretical Approaches and Previous Findings

In the aftermath of the economic and financial crisis that started in the fall of 2007, several studies reported declining fertility rates in the economies hit by the Great Recession (Goldstein et al. 2013; Lanzieri 2013; Hillamo 2017; Örsal and Goldstein 2018; Seltzer 2019). They suggest that birth rates tend to decline in association with negative macroeconomic developments, such as declining economic growth, labor-market insecurity, and increasing unemployment. In addition, these developments occurred at the same time as changes in less-tangible measures of actual and perceived economic uncertainty, such as the rise in private and public debt and housing-foreclosure rates, the reduction in consumer confidence, and the negative tone in the media coverage of a crisis (Schneider 2015; Comolli 2017). Although crisis-related declines in fertility are usually smaller and more temporary compared to persistent long-term secular demographic trends (Lee 2003; Lesthaeghe 2010), strong recessions can be followed by long-term fertility declines (Elder 1974; Comolli and Bernardi 2015).

Why do some people postpone or forgo childbearing during a recession, and why may recessions have long-term effects on childbearing behavior? We sketch several theoretical answers: First, economic theories assume that the *income effect* (Becker 1960) is at play. It states that couples avoid expensive, irreversible, and life-changing investments such as having a child when the expected future income level is low.^1^ Second, recessions may have negative effects on partnership and marriage formation and stability (Mills and Blossfeld 2003). Financial strain and poor economic prospects may cast doubts on one’s ability to provide for a family—a mechanism Oppenheimer (1994, 1997) referred to as *uncertainty effect* to distinguish it from the purely financial income effect described above. Fertility rates may therefore decline during a recession due to the depressed rates of entry into co-residential unions and marriage and the higher rates of separation and divorce (Fishback et al. 2007; Jalovaara and Fasang 2017; Miettinen and Jalovaara 2019). Oppenheimer’s uncertainty dimension relates to how one’s own future prospects are evaluated on the basis of current circumstances such as educational attainment, employment status, and job type. A third mechanism, the *perceived uncertainty effect,* or *wait-and-see,* is not necessarily based on one’s factual financial or labor-market situation. The perception of uncertainty may go far beyond the material economic conditions in which individuals currently live and relate to their “narratives of the future” (Vignoli et al. 2020), namely how they imagine and assess their future. The mechanism is twofold. First, according to Bloom (2009), the economic uncertainty generated by major recessions makes actors “wait and see” how the future develops before making any far-reaching decisions. Second, Comolli (2017) and Schneider (2015) show that perceived uncertainty—although more difficult to measure—raises the insecurity about future financial and labor-market opportunities and induces individuals to abstain from or postpone having children. Childbearing may therefore be postponed in the wake of a recession, even if the economy has recovered.

Fourth, as a new aspect, we argue that the welfare state also plays a role. We think that welfare-state retrenchment or social-policy expansions during an economic crisis may also influence childbearing behavior. Roosevelt’s New Deal and the reversal of the fertility decline in the USA during the Great Depression is a first example of the link between reform programs that support and protect people at large, and fertility development during an economic trough (Fishback et al. 2007). In welfare states with comprehensive coverage of social, family, and labor-market risks, such as the Nordic and many other European welfare states, childbearing decisions may depend on the capacity of the social-security system to provide the protection and services that people expect to potentially need. Cuts to benefits and services, and investments in employment and education, enacted in order to tackle an economic crisis send countervailing signals as to how much people can rely on the welfare state to protect them in times of uncertainty and to maintain their own and their families’ well-being in the future. Beyond the policy measures taken, people’s perceptions of their current and future well-being may also depend on whether the crisis is perceived as a national one that can be managed by the nation state or as a global crisis that may limit the nation state’s capacity to tackle it in the short and the long run. We term the mechanism that links the welfare state and crisis management to fertility the *perceived welfare uncertainty*.

Regarding the Nordic countries, researchers have mostly studied the business cycle–fertility nexus separately for each country (Andersson 2000; Kravdal 2002; Vikat 2004; Huttunen and Kellokumpu 2016; Jónsson 2018). Their findings are usually in accordance with those concerning other advanced economies. In the Nordic countries, the decline in births in response to economic downturns tends to be concentrated among young adults and at lower parities (first and second birth). Jónsson (2018) reports that in Iceland, after the onset of the economic crisis in 2008, a trend of declining first-birth intensity emerged. In 2011, three years into the crisis, turnarounds also occurred in second- and third-birth rates, which continued until the end of the study period in 2013. Jónsson argues that simultaneous decrements in parental-leave benefits were partially responsible for the post-crisis decline in birth intensities and that family policies failed to compensate for the impact of the economic crisis on fertility. For Sweden, Andersson (2000) demonstrates that the increasing share of women with low income and enrolled in education during the 1990s crisis explains much of the decline in fertility during that period. Also studying Sweden, Hoem (2000) shows that women’s first-birth rates correlate with their municipalities’ employment rates, especially regarding births to young women below the age of thirty. For Finland, Hiilamo (2017) finds a negative association between unemployment and fertility rates during 1991–2015. However, Comolli (2018) shows that if the two periods of the 1990s and 2000s are considered separately, a reversal from a positive to a negative association between unemployment and fertility emerges in Finland. Comparing childbearing rates in the Nordic countries, Neyer et al. (2006) attribute the differences in fertility development in Sweden and Finland after the 1990s crisis to different family policies in these countries. Finally, Dommermuth and Lappegård (2017) investigate the Norwegian context, where the most recent drop in TFR seems to be due both to a change in the timing of first births and a long-term negative trend in women’s likelihood of having three or more children. Economic activity, work experience, and the unemployment rate in the municipality significantly affected first-birth rates. The decline in third births is instead a reflection of a long-lasting downward trend, although results also suggest that individual income and local unemployment rates have had an impact on the continued decline in third-birth rates that has taken place since 2010.

A few comparative studies on Europe also include the Nordic countries. Neels et al. (2013) focus on first-birth risks in fourteen countries, including four Nordic countries (excluding Iceland). They show that periods of deteriorating economic conditions during 1970–2005 significantly reduced first-birth hazards, especially among men and women below age thirty. Goldstein et al. (2013) do not find a significant aggregate fertility response to unemployment rates in the Nordic-country cluster (excluding Iceland) during the recent Great Recession, except for the early twenties age group. However, Comolli (2017) suggests that when considering more recent years after the onset of the crisis in Europe, the impact on fertility is also larger than previously thought for the Nordic countries.

## Data and Method

We use high-quality, harmonized population-register data on all women born in Denmark, Finland, Iceland, Norway, and Sweden. The data offer full, accurate, and reliable coverage of the entire population in each country and their vital events, including the entire childbearing histories of our subjects. We focus on the native-born population of women to ensure that we dispose of their entire childbearing and educational histories. We follow women born in 1942–2003, with a focus on childbearing risk during the period ranging from the late 1980s to the mid-2010s. Table 1 shows the initial size of our study population, i.e., the pool of women at risk of first birth, for each dataset and the calendar periods for which they are available.

**Table 1:** Number of women at risk of first birth and period of observation

Country	N	Period
Finland	1,382,230	1987–2017
Denmark	1,352,104	1987–2017
Norway	1,187,802	1987–2017
Sweden	1,976,281	1987–2017
Iceland	70,294	1987–2013

*Source*: Elaboration of the authors based on each country’s register data

We use event-history techniques to present parity-specific indices of childbearing risks over calendar years relative to a baseline year. Calculations are based on the records of registered live births in each country and the corresponding exposure times at risk, by birth order. Women enter the analysis in the month they turn sixteen for parity one, and at the time of last previous birth for higher-order parities. For each birth order, we present relative risks of childbirth for each country and calendar year, standardized for age and duration since previous birth, which means that we control for the effect of compositional demographic changes over the categories of these variables over time. We censor observations at age forty-six, first outmigration, first twin birth, death, and end of observation period. This method allows us to examine the period effect of the economic crises at the individual level. The approach is described further in Hoem (1991, 1993).

We focus explicitly on the period effects of the two recessions by presenting separate time series of relative risks for women in two periods: 1987–1999 and 2003–2017. Each period has a reference year (1990 and 2008, respectively) as the baseline year from which the deviation in the relative risk is measured. By applying event-history techniques to the population-register data of five countries, we are able to get an accurate picture of similarities and differences in childbearing responses to the two episodes of recession in the countries considered. We thus investigate 5 × 2 country-period cases of childbearing dynamics before, during, and after the crises, running separate but identical models for each country. Our focus is on relative changes in fertility levels of each country in the wake of each recession rather than differences in absolute levels of fertility between countries that belong to the same Nordic fertility regime (cf. Andersson 2004; Neyer et al. 2006). The contribution of our approach is that we can analyze these country-specific relative changes separately by parity, education, and age in a regression framework. We present the trends in relative risks for the years that preceded the onset of each crisis to offer additional evidence on the relative changes that occurred subsequent to these crises.

Indeed, we suspect that changes in the childbearing intensity during the two recession episodes have been heterogeneous across sociodemographic groups. We thus run separate analyses for each childbearing parity, including higher-order ones up to the fourth birth. We further distinguish the standardized birth rates by age group (16–29 versus 30–46 years) and educational level: primary (including lower-secondary level), secondary (upper-secondary level), and tertiary education. Results are mainly presented graphically by parity, age group, and educational level to facilitate the cross-country comparison. Full tables of relative risks are available in Appendix.

## Results

### Birth Risk by Parity

**Fig. 2:**
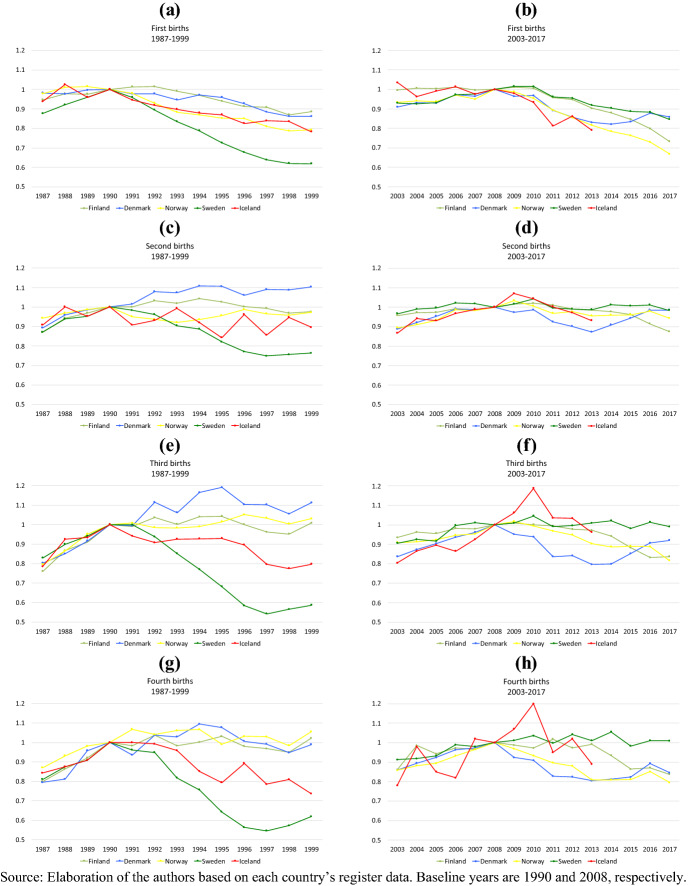
Relative risks of birth by parity and period (1987–1999 and 2003–2017)

Figure 2 displays the relative risk of the first to fourth births, by country, in the two periods (panels a–h). The panels on the left-hand side show the yearly risks of births in the period 1987–1999 relative to the baseline year 1990, while the right-hand-side panels show the relative risks in the period 2003–2017 relative to year 2008. Before the onset of both recessions, relative risks of childbirth showed a stable or mildly increasing trend in all countries, with the most positive trends observed for the higher parities. In relative terms, the childbearing pre-crises trends were quite homogenous in the five countries.

**Fig. 3:**
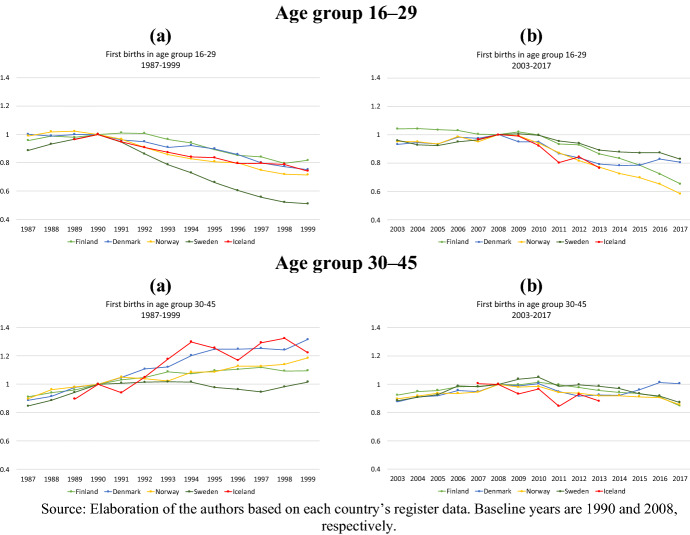
Relative risk of first births by Period (1987–1999 and 2003–2017) and age group

In all Nordic countries, the relative risk of first birth declined in the years following the recession onsets, and this happened in both recessionary periods. Relative to 1990, the strongest drop in the risk of first birth was witnessed in Sweden, followed by Iceland and Norway. A delayed and milder, but still observable, reduction in the risk of first birth occurred in Denmark and Finland. Ten years after the onset of the crisis, Sweden still had a relative risk of first birth 40 percent lower than in 1990. In the other four countries in 1999, the relative risk of first birth was around 10–20 percent lower. (Some of this is due to postponed first-birth fertility; see next in Fig. 3.) The drop in the relative risk of first birth was only slightly smaller in the more recent period. Norway then experienced the largest drop, as seen over the entire follow up (around -35 percent in 2017 relative to 2008) closely followed by Finland where the decline accelerated after 2015 (almost -30 percent in 2017 relative to 2008). Iceland and Denmark witnessed a similar decline as Norway early into the recession, but contrary to Norway, the relative risk of first birth in Denmark started recovering after 2014.^2^

The change in the relative risk of second birth is more heterogeneous across the two periods. Relative to 1990, the risk of second birth declined more pronouncedly in Iceland and Sweden than in the other Nordic countries; it dropped but quickly recovered in Norway, remained basically the same in Finland, and increased in Denmark. By contrast, relative to 2008, the risk of second birth did not change much. One exception is Denmark, where in 2013, the risk of a second birth was more than 10 percent lower than before the onset of the Great Recession, but it recovered after 2013 to reach pre-crisis levels in 2016. In Finland, the relative risk of second birth also dropped by some 10 percent, but only after 2015.

The figures for third and fourth parities are extremely similar to each other within each period but again differ strongly across the two periods. First, the period changes in relative risks were more heterogeneous in the 1990s than after 2008. Relative to 1990, the risk of higher-parity births dropped most dramatically in Sweden (more than − 45 percent), followed by Iceland. In comparison, after 2008, the same two countries, if anything, witnessed an increase in the relative risk of higher-order births. In Denmark, the opposite held, with higher-order fertility increasing after 1990 and decreasing after 2008. Finally, in Norway and Finland there was basically no change in the risk of third and fourth births in the 1990s, while a drop was observed in third births in both countries, beginning in 2013 in Finland. An analogous drop in fourth births after 2013 was evident in Finland, while in Norway, the decline started much earlier.

Overall, we can conclude that, as found in previous studies, lower parities were more uniformly negatively affected by economic conditions across periods and contexts (Goldstein et al. 2013; Comolli 2017). More importantly though, despite the uniformity of pre-recession trends, the drops in the relative risk of childbearing after 1990 were far more heterogeneous across the Nordic countries than after the Great Recession. After 2008, the decline in childbearing risk was milder but more persistent and homogenous across the five countries.

### Birth Risk by Parity and Age Group

Figure 3 shows the two periods’ changes in relative risks of first birth by women’s age groups: 16–29 versus 30–45 years. The substantive impressions from the results regarding higher parities are very similar to those presented in Figs. 2 and 3, and the corresponding figures by age groups for higher parities are reported in Appendix (“Appendix Figs. 6, 7”). Overall, we see again that after the onset of the Great Recession, the variation in changes in childbearing risks was more homogenous across countries than in the 1990s, especially among women aged 30 and older and at higher parities (cf. “Appendix Figs. 6,7”). During the later period, relative first-birth risks varied in the range of 0.60–0.80 among the younger and 0.85–1 among the older women, while during the earlier period, they varied in the range 0.50–1 and 1–1.30, respectively. In all five countries, the strongest decline in the relative risk of first birth took place among women younger than thirty. By 1999, the relative risk of first birth for young Swedish women was 50 percent lower than in 1990. In all other countries, it was more than 20 percent lower. In the same age group, the risk of first birth in 2017 was around 40 percent lower than in 2008 in Norway, around 35 percent lower in Finland and between 10 and 20 percent lower in Denmark and Sweden.

Further, we observe a major difference in the developments of birth risks of older women between the two periods. In the 1990s, notwithstanding the onset of the crisis, the relative risk of first birth among women aged 30 and older remained constant in Sweden and even increased in the other countries, continuing the pre-1990 positive trend. Five years into the crisis, the relative risk of having a first child was 25 percent higher in Iceland and Denmark and 10 percent higher in Norway and Finland, and it stayed similarly high until the end of the 1990s, altogether reflecting postponed first-birth fertility. After 2010, in contrast, the pre-crisis trend of increasing relative first-birth risks of women aged 30 and older came to an end and the relative risk of first birth declined among older women too, contributing to the overall decline in the quantum of fertility. The decline among older women, though, was milder compared to that among younger women, with declines of about 10–15 percent in most countries. Only in Denmark did the relative risk of first birth among women aged 30–45 recuperate to pre-2008 levels in 2016–2017.

The divergence in first-birth risks between younger and older women in the 1990s is largely a result of increasing ages at becoming a parent during that period. Women in their twenties postponed childbearing, which was recuperated by women in their thirties. It is interesting to note, though, that even during the 1990s economic crisis, women over thirty kept recuperating childbearing. In contrast, in the more recent period, which was also characterized by declines in the younger age group, recuperation among women in their thirties was less evident. In most countries during this period, birth rates in the older age bracket declined as well.

### Birth Risk by Parity and Educational Level

**Fig. 4:**
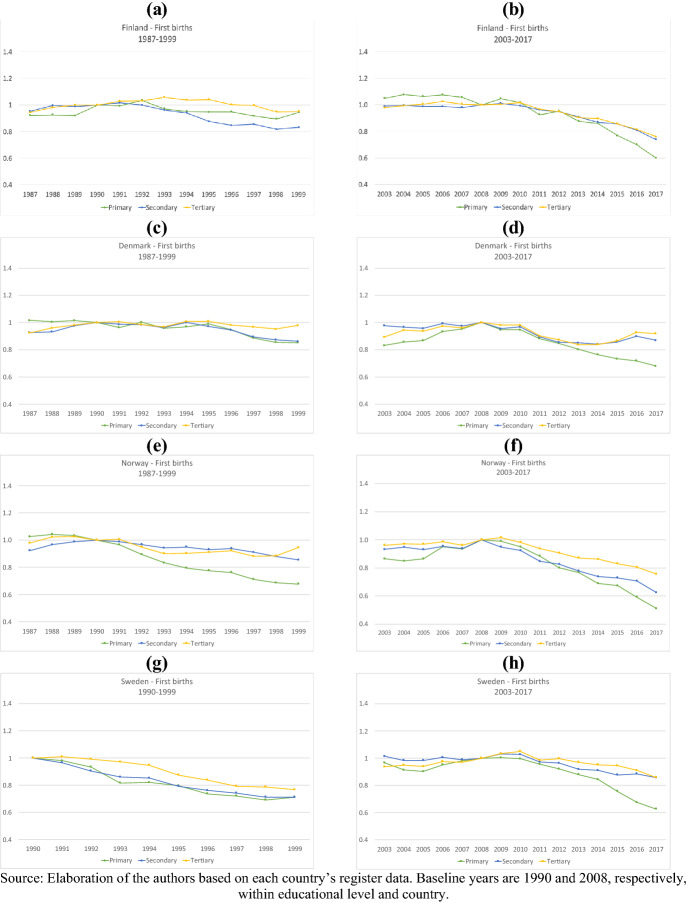
Relative risk of first births by country, education, and period (1987–1999 and 2003–2017)

**Fig. 5:**
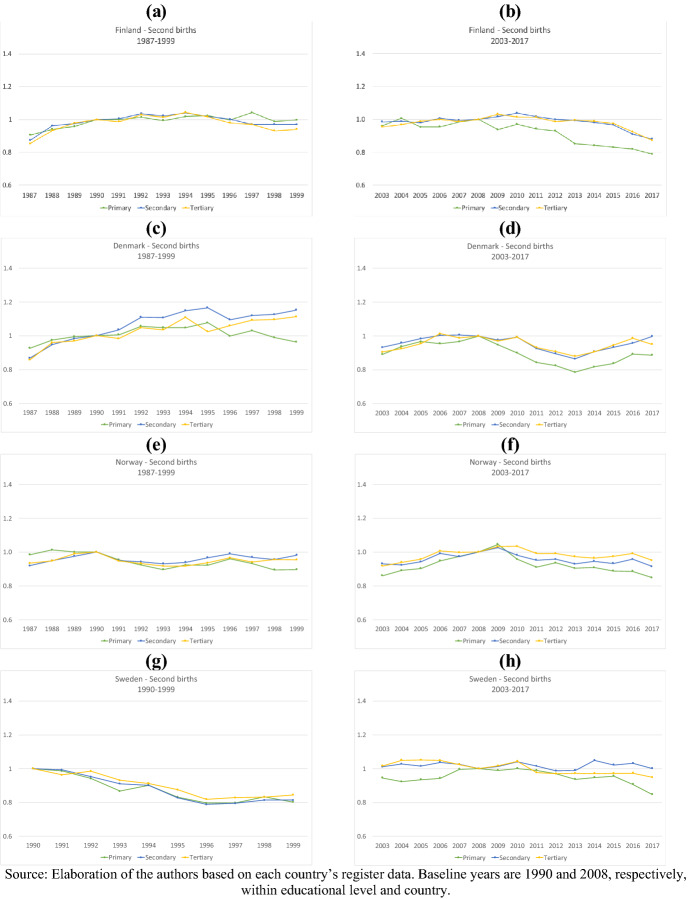
Relative risk of second birth by country, education, and period (1987–1999 and 2003–2017)

**Fig. 8:**
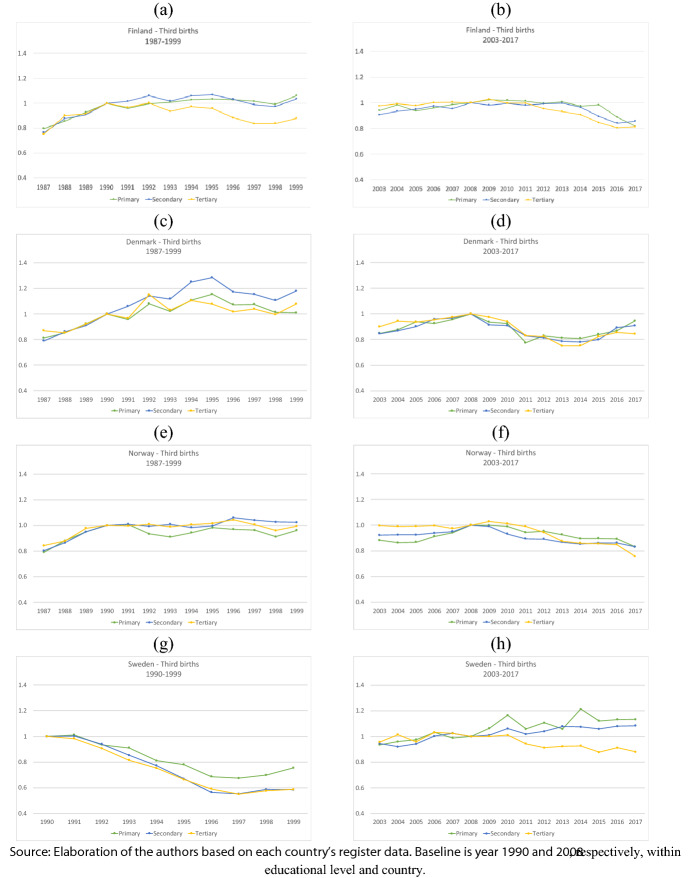
Relative risk of third births by country, education, and period (1987–1999 and 2003–2017)

**Fig. 9:**
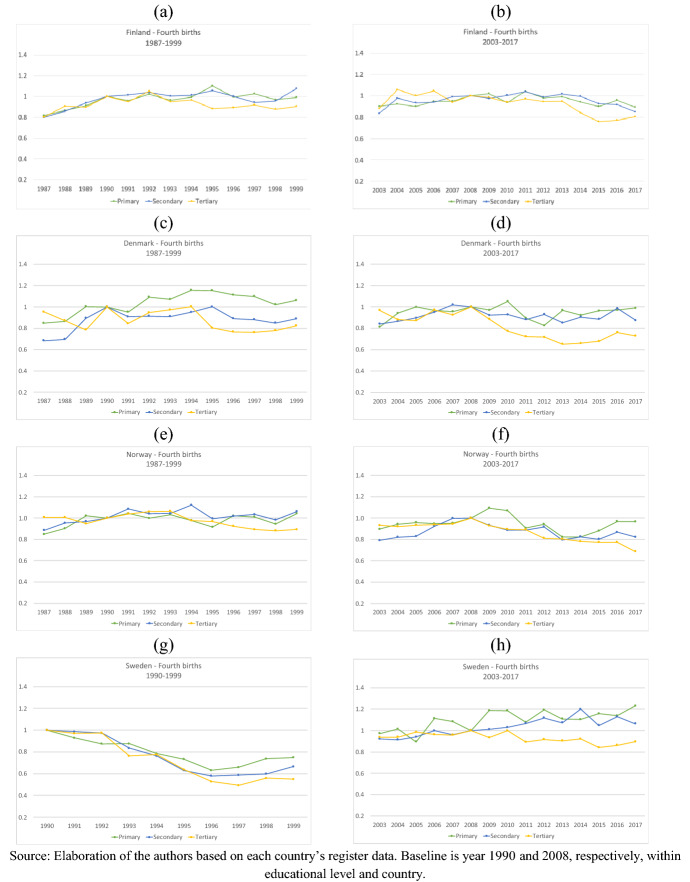
Relative risk of fourth birth by country, education, and period (1987–1999 and 2003–2017)

Figures 4, 5 show the relative risk of first and second births in the two periods by women’s levels of education. To facilitate the comparison across educational groups, results are plotted by country, and the reference category is the risk of first or second birth in the baseline years (1990 and 2008) within each educational group. For Iceland, unfortunately, data on education were not available, and therefore, it had to be excluded; for Sweden, data on education are available from 1990 onwards only. In many aspects, higher parities offer similar findings to those presented for parities one and two across educational groups, and the results are reported in Appendix (Figs. 8, 9).

First, we notice that in each country, the development of the relative risks of first births was more education-specific in the 1990s than after 2008, at least until 2014. In Sweden in the wake of the 1990s crisis, first-birth risks declined in all educational groups but more pronouncedly among lower-educated women than among women with higher education. Similarly, in Norway, first-birth risks in the 1990s declined markedly among low-educated women, but contrary to Sweden, the decrease in first-birth risks among secondary and tertiary-educated women was much more moderate and rather similar. In Denmark, first-birth risks among women of all educational groups remained relatively stable and remarkably similar during the first half of the 1990s. Thereafter, the relative risk of first birth declined somewhat among medium- and low-educated women. In Finland, the largest drop in relative risks of first birth was found among the medium-educated women, followed by a more moderate decline among low-educated women. The first-birth risks of tertiary-educated women in Finland even slightly increased in the early 1990s. As Fig. 4 shows and as confirmed by previous studies (Vikat 2004; Rønsen 2004), the positive trend in the fertility among highly educated Finnish women started during the 1980s, but it is still remarkable to see that the onset of the recession did not halt the rise in first-birth risks.

Compared to the 1990s, first-birth risks in the initial years after 2008 developed more similarly across all educational groups and all countries: First-birth risks dropped to a similar extent for all categories of women. In Finland and Denmark, there was no difference at all in the development of first-birth risks between the different educational groups between 2008 and 2013. In Norway and Sweden, the decline in first-birth risks was more substantial among less-educated women but not remarkably so until 2015 (Fig. 4). After 2014, the uniformity ends. In all the four countries, the relative risk of first births among primary educated women diverged from those of the other two educational groups. Compared to 2008, in 2017 the relatives risks for women with only primary education were 15 percentage points lower than the corresponding relative risks for women with tertiary education in Finland, around 25 percentage points lower in Sweden and Norway, and 30 percent lower in Denmark.

For second births, the educational differences in the development of relative risks in the 1990s were smaller than for first births in all countries except Denmark. Only in Sweden did the relative risks of second births decline noticeably in all educational groups (Fig. 5). In Finland, similar to first-birth risks, second-birth risks among highly (and here also medium) educated women were increasing in the first few years following the 1990s crisis. The development of second-birth risks after the Great Recession was quite uniform among the educational groups. However, as with parity one, the relative risk of second births among the low educated declined at a faster rate than for others. Finally, while we find no huge educational differences in the developments of third-birth risks, in both periods, tertiary-educated women registered a steeper decline in the relative risk of a fourth birth than upper-secondary or primary-educated women (Figs. 8, 9).

Overall, we find that regarding first births, highly educated women were least affected by the 1990s crisis. Their first-birth risks slightly increased (Finland), remained stable (Denmark and Norway), or declined much less (Sweden) than the first-birth risks of low- and medium-educated women. In the first years after 2008, such educational differences in the development of first-birth risks no longer existed; the relative risks of entering parenthood declined at the same pace in each educational group. However, after 2014 primary educated women register a steeper continued drop in their relative risk of becoming a mother. We further notice that while in the 1990s, it was primarily the behavior of tertiary-educated women that differed from the others (in positive terms), after 2014 it was the childbearing of primary educated women that deviated (in negative terms) from those of the mid and high educated. With respect to second births, educational differences after 1990 were only visible in Finland and Denmark. After 2008, the development of second-birth risks across educational groups was more homogenous in all countries, but primary educated women displayed a more negative trend. Only at very high parities did the educational gradient in period changes reverse and tertiary-educated mothers displayed the steepest declines in relative risks of childbearing.

## Discussion

The decline in the Nordic countries in period total fertility after 2010 has caught many scholars by surprise and attracted the attention of the media and policy makers in the region. The timing of the fertility-rate contraction suggests a link with the global economic and financial crisis that plagued most advanced economies in 2008 and some years thereafter. Research has shown that fertility rates in advanced economies are pro-cyclical, so they tend to rise with economic growth and decline with stagnation or recession (Sobotka et al. 2011; Comolli 2017; Örsal and Goldstein 2018). The cyclicality of fertility rates in the Nordic region has not been systematically studied in comparative terms prior to this study, neither across the five Nordic countries nor across the two economic crises that hit the region during the last decades. However, single-country studies have demonstrated that the Nordic countries are not exceptional with regard to the pro-cyclicality of birth rates (Andersson 2000; for Finland’s singularity see Comolli 2018). The main theoretical assumptions for a pro-cyclical behavior state that economic and financial uncertainty and labor-market insecurity—usually measured through declining or negative GDP growth rates and rises in unemployment rates—induce individuals to delay major life commitments such as family formation. Childbearing is a costly and irreversible transition, and even if couples do not directly experience a drop in income (*income effect*) or joblessness (*uncertainty effect*), they may perceive their futures as less secure if the economy slows down or skids into a recession (*perceived economic uncertainty effect*). In response, couples may postpone childbearing during a recession, and prefer to *wait-and-see* (Bloom 2009; Bachmann and Bayer 2013) how the economy evolves before having a(nother) child.

In our study, we focus on the development of childbearing during a crisis, but even more so on the development *after* a crisis. We compare country-specific annual childbearing risks relative to a baseline at the onset of two economic critical junctures (i.e., the 1990–1992 and the 2008–2010 recessions) in each Nordic country. Our systematic comparison of parity-, age- and education-specific childbearing risks across two crises and five “similar” welfare states allows us to reflect on existing theories and contemplate neglected theoretical links that may be pursued empirically in future research. Our empirical presentation was mainly descriptive, and we did not provide the rationale for empirical tests of different theoretical hypotheses. We leave for future research to, for example, evaluate the differential role of long-term family change, macroeconomic factors and labor-market restructuring, individual sociodemographic circumstances, perception of uncertainty, and policy changes during recession episodes to disentangle the extent to which the decline in fertility may be related to each component.

The study produced several thought-provoking findings. Finland’s fertility increase among highly educated women during the 1990s mass unemployment is interesting. The countercyclical tendencies in Finland’s fertility during the 1990s recession have been shown elsewhere (Vikat 2004; Neyer et al. 2006; Comolli 2018). Here, we add that they were most pronounced among tertiary-educated women. These women are better established in the labor market, have more secure employment contracts, higher earnings, and more often live in a stable partnership, but may have postponed births due to career entry, for instance. All of these factors were conducive to their childbearing during the crisis in the 1990s (Vikat 2004; Comolli 2019). The extension of public childcare and the increase in the home-care allowance during the first years of the crisis provided an additional safety net that also covered women with less education and a more fragile position in the labor market (see also Vikat 2004).

More generally, although the five Nordic countries have shared similar macroeconomic outlooks during the last half century, with similar labor-market structures, women’s employment rates, welfare-state orientation, and, as we have shown, homogenous trends in fertility rates in the late 1980s, there was significant country heterogeneity in the fertility developments after the 1990s recession. Moreover, even though in most Nordic countries, the more recent crisis in 2008 was milder and lasted less long than that in the 1990s, the latter seems to have had more persistent and uniform scarring effects on childbearing intensities across countries, parities, ages, and educational groups. Further, our findings show that the decline in first birth rates accelerated in the second half of the 2010s in three out of four countries for which we have data for these years, and, that first and second birth rates to low-educated women dropped at an increasing rate in these more recent years.

How do these findings relate to theories about the link between economic crises and fertility? First, the differences in childbearing outcomes between the two crises suggest that one crisis is not the same as another. Second, a purely economic perspective that focuses on factual income reduction and financial insecurity is too narrow to explain the differential patterns of fertility development that we find for the 1990s and the 2010s. We need a broader framework that incorporates additional perspectives beyond the strictly economic ones and that better contextualizes each crisis. Reflecting on our findings, we suggest two dimensions for extending this framework: First, we need to consider how countries and welfare states manage an economic crisis and how this may be related to subsequent fertility behavior. This *crisis management*—that is, the policies that are introduced to tackle economic turmoil—may be directed at the economic performance of the country—for example, by stabilizing the banking sector or supporting specific industries. However, crisis management may also involve amendments of labor-market, social, and family policies (see also Sobotka et al. 2011; Fishback et al. 2007). For example, our study results for the 1990s suggest that whether a country extends or cuts its social and family policies during an economic crisis may influence childbearing behavior during and after it. Such welfare amendments may be particularly relevant for fertility in developed welfare states, where markets are more regulated by policies (Hall and Soskice 2001) and social policies better guard against market risks than in, for example, liberal economies. Individuals in comprehensive welfare states may rely more on the protective capacity of the state for their support during a crisis (Kumlin et al. 2018).

How countries handle a crisis and what policies are implemented in its aftermath may not only influence individuals’ factual livelihood, but also their perception of uncertainty—of economic insecurity as well as *perceived welfare uncertainty*, i.e., their trust in the present and future ability of the welfare system to protect them against economic and life-course adversities and secure their own and their offspring’s well-being (see also Mau et al. 2012). Hiilamo (2019) suggests as one of the causes of the 2010s fertility decline in Finland that policies to support the work-family balance have not matched women’s expectations during that decade. A similar suggestion is offered by Jónsson (2018) for Iceland.

How countries handled the 1990s crisis and how individuals perceived their economic and welfare security during that time may be relevant elements behind the differences in fertility developments that were observed during that decade. To a large extent, the 1990s crisis was unexpected, due to idiosyncratic shocks, and each country handled it largely nationally following very different approaches: Sweden cut its benefits considerably (after expanding its welfare state in the 1980s); Finland reacted less severely and less abruptly; and Denmark expanded its parental-leave program and introduced job-rotation programs, while at the same time imposing shorter durations and stricter eligibility criteria for receiving unemployment benefits. Subsequently, all five Nordic countries mobilized and extended their active labor-market programs (albeit with different strengths) and supported education programs for young people, particularly those with lower levels of training.

In 2008, the situation was different. The 2008 crisis also came unexpectedly. However, it had a common external cause and it was handled more quickly and less idiosyncratically with respect to social policies. Furthermore, the countries concerned could rely on the lessons learned during the 1990s (Ólofsson et al. 2019). Except in Iceland, the crisis was also less severe and shorter than the previous one. The greater homogeneity in handling the recession seems to have generated a more uniform fertility response across countries in the initial years following the recession. This can be observed independently of parity, age, and educational level. After 2010, even without an ongoing recession most of the Nordic countries have calibrated their social spending and tightened access to social benefits. This tends to affect the lower strata of the population more than others who are less dependent on social support. The steeper drop in first and second birth rates that was observed among low-educated women after 2014 might be an unintended consequence of the inequality produced by the combination of the recession and the post-crisis welfare cuts.

Welfare retrenchment may be one component behind the strength and persistence of the drop in childbearing intensities for the low educated in the second half of the 2010s. However, welfare restrictions are as unsatisfactory an indicator as that of macroeconomic indexes in explaining why childbearing propensities have declined among groups that are not affected by tightened welfare support, such as the medium and highly educated. Moreover, other Western countries have experienced similar fertility declines, sometimes starting a few years earlier than in the Nordic countries. In the USA and other countries in Southern and Western Europe, the fertility declines persist and, at the time of writing (2020), there have been no signs of trend reversals. The same goes for the five Nordic countries examined in this study. The magnitude of the fertility rate contraction in the USA is very similar to that of the Nordic countries, with a TFR declining from 2.12 in 2007 to 1.73 children per woman in 2018 (CDC 2019). This similarity in fertility decline across many, but not all, developed contexts calls for expanding our explanatory framework by a second perspective that goes beyond purely economic and state-related dimensions: that of *perceived global uncertainty.*

Although demographers often view national fertility developments in the context of the nation state, such a confinement may no longer be suitable. Today’s world is much more interconnected than in the early 1990s, economically as well as socially. The availability of mass communication channels that were not yet established in the early 1990s, such as smartphones and social media, may increase the perception that the future bears unknown uncertainties that cannot be controlled locally or nationally. Global changes in production or economic turmoil elsewhere tend to affect national economies and labor markets more than before, while the capacity of states to influence such global events have become more limited. Similarly, other events that happen internationally, e.g., terror acts and armed conflicts, and developments that cannot be contained nationally, such as climate change or pandemics, may increase people’s perception of uncertainty in a global perspective. At the time of the writing (2020), this becomes clearly evident as the ongoing COVID-19 pandemic is not only a global health crisis, but most expert also expect a new long-lasting global economic crisis, despite different national investment programs and social-policy measures.

We do not know the extent to which subjective factors of this kind may lie behind the surprisingly uniform 2010s fertility decline in the Nordic countries despite the countries’ underlying steady material circumstances and the stability of the Nordic welfare state. A study on regional fertility trends in Europe by Matysiak et al. (2020) also highlights the Nordic countries as a case where structural factors related to economic indicators seem particularly ill-suited to explain recent and ongoing fertility change. To address many of these issues, we need to rely on data that also cover different aspects of people’s subjective considerations of their life situation and the uncertainties they may perceive. A new round of data collection in the Generations and Gender Programme will help in this regard. Meanwhile, our findings warrant to revise theoretical assumptions about the effects of economic crises on fertility behavior. To view childbearing outcomes after a crisis from a broader perspective that incorporates economic and welfare state issues as well as individual perceptions of—economic, welfare, and global—uncertainties may help piece together the puzzles regarding post-crisis and current fertility declines. We thus conclude that future research on the links between economic recessions and fertility development needs to expand the explanatory framework considerably, and that much theoretical and empirical work is still to be done to fully grasp the relationship between economic cycles and fertility outcomes. With the expected economic crisis and other social developments that unfold in the wake of the 2020 COVID-19 crisis, much future research of this kind is anticipated.

## Figures and Tables

**Fig. 6:**
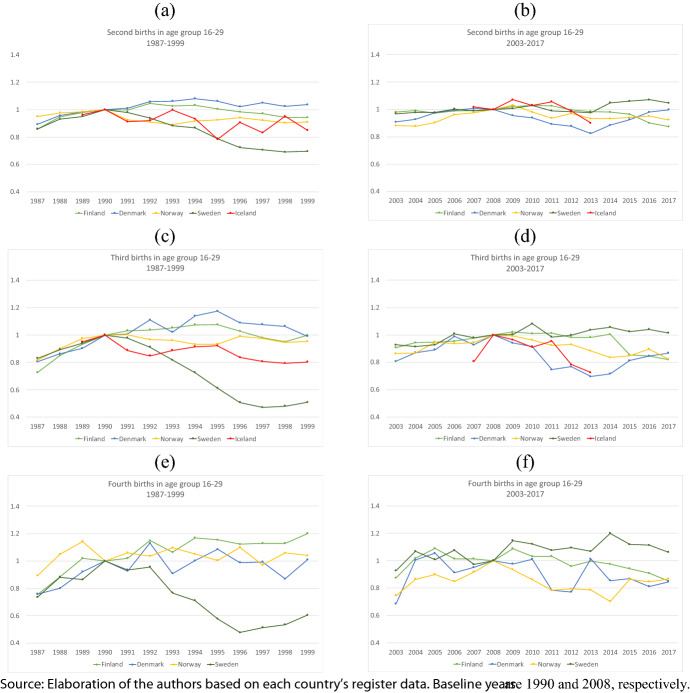
Relative risk of births by parity and period (1987–1999 and 2003–2017). Second, third, and fourth parities. Age group 16–29

**Fig. 7:**
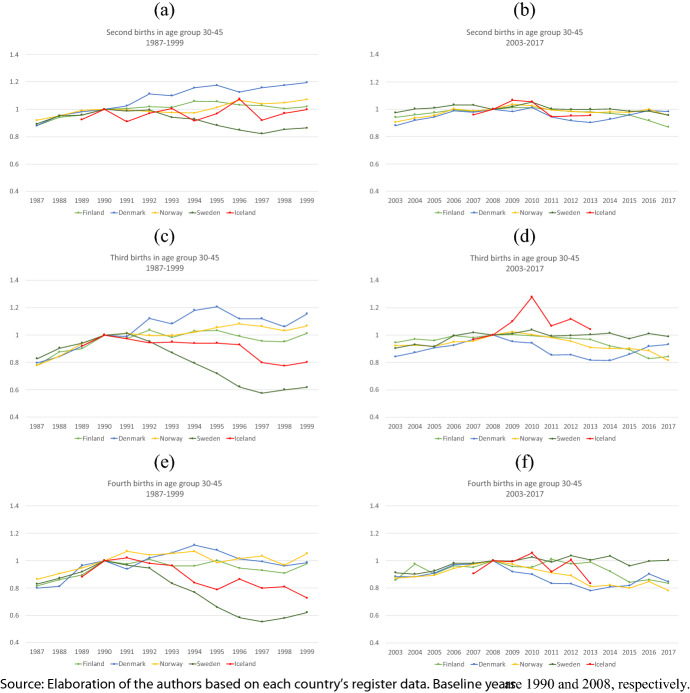
Relative risk of births by parity and period (1987–1999 and 2003–2017). Second, third, and fourth parities. Age group 30–45

## Appendix

See Figures 6, 7, 8, and 9.

See Tables 2, 3 , 4, 5 , 6, and 7.

**Table 2:** Aggregate indicators for each country, 1970–2019

	GDP growth (annual %)					Unemployment rate					Total fertility rate				
	FI	DK	IS	NO	SE	FI	DK	IS	NO	SE	FI	DK	IS	NO	SE
1970	5.33	2.74	9.37	2.00	6.04	2.0	1.0		1.6	1.5	1.83	1.95	2.81	2.50	1.92
1971	2.36	3.01	13.06	5.67	0.94	2.4	1.3		1.5	2.5	1.68	2.04	2.92	2.49	1.96
1972	7.74	3.93	6.18	5.33	2.29	2.7	1.4		1.7	2.7	1.58	2.03	3.09	2.38	1.91
1973	6.98	4.09	6.81	4.53	3.97	2.4	0.9		1.5	2.5	1.49	1.92	2.94	2.23	1.87
1974	3.24	− 1.12	5.71	3.92	3.20	1.8	2.1		1.5	2.0	1.61	1.90	2.66	2.13	1.87
1975	1.80	− 1.46	0.65	4.95	2.55	2.6	5.1		2.3	1.6	1.68	1.92	2.65	1.98	1.77
1976	0.34	5.92	5.96	5.83	1.06	3.9	5.3		1.8	1.6	1.70	1.75	2.52	1.86	1.68
1977	0.24	1.87	8.82	4.16	− 1.60	5.9	6.4		1.5	1.8	1.68	1.66	2.31	1.75	1.64
1978	2.92	2.23	6.02	3.87	1.75	7.3	7.3		1.8	2.2	1.64	1.67	2.35	1.77	1.60
1979	7.12	3.87	4.86	4.37	3.84	6.0	6.1		2.0	2.1	1.64	1.60	2.49	1.75	1.66
1980	5.39	− 0.48	5.75	4.56	1.70	4.6	4.9	0.3	1.6	2.0	1.63	1.55	2.48	1.72	1.68
1981	1.29	− 0.67	4.27	1.60	0.45	4.8	7.9	0.4	2.0	2.5	1.64	1.44	2.33	1.70	1.63
1982	3.09	3.68	2.15	0.24	1.25	5.3	8.4	0.7	2.6	3.2	1.71	1.43	2.27	1.71	1.62
1983	3.12	2.60	− 2.15	3.97	1.90	5.4	8.4	1.0	3.4	3.7	1.74	1.38	2.24	1.66	1.61
1984	3.21	4.17	4.13	6.05	4.23	5.2	7.9	1.3	3.1	3.3	1.69	1.40	2.08	1.66	1.66
1985	3.54	4.00	3.29	5.55	2.16	5.0	6.7	0.9	2.6	2.9	1.64	1.45	1.93	1.68	1.74
1986	2.73	4.90	6.27	4.04	2.69	6.7	5.0	0.6	2.0	2.7	1.60	1.48	1.92	1.71	1.80
1987	3.56	0.25	8.55	1.75	3.35	4.9	5.0	0.5	2.1	2.2	1.59	1.50	2.06	1.74	1.84
1988	5.21	− 0.01	− 0.09	− 0.26	2.56	4.2	5.7	0.6	3.2	1.8	1.69	1.56	2.26	1.84	1.96
1989	5.09	0.65	0.26	1.04	2.65	3.1	6.8	1.6	4.9	1.6	1.71	1.62	2.19	1.89	2.01
1990	0.68	1.48	1.17	1.93	0.75	3.2	8.5	1.8	3.5	1.6	1.78	1.67	2.30	1.93	2.13
1991	− 5.91	1.39	− 0.22	3.08	− 1.15	6.7	9.2	2.7	3.6	3.0	1.79	1.68	2.18	1.92	2.11
1992	− 3.32	1.96	− 3.37	3.57	− 1.16	11.8	9.2	4.4	3.9	5.2	1.85	1.76	2.21	1.88	2.09
1993	− 0.73	0.01	1.31	2.85	− 2.07	16.5	10.9	5.3	4.6	8.2	1.81	1.75	2.22	1.86	1.99
1994	3.94	5.33	3.61	5.06	4.09	16.7	8.1	5.4	4.5	8.0	1.85	1.81	2.14	1.87	1.88
1995	4.21	3.03	0.12	4.15	4.02	15.5	7.0	5.0	4.7	7.7	1.81	1.80	2.08	1.87	1.73
1996	3.66	2.90	4.79	5.03	1.52	14.7	6.9	3.7	3.5	8.0	1.76	1.75	2.12	1.89	1.60
1997	6.25	3.26	4.91	5.28	2.90	12.7	5.4	3.8	2.6	8.0	1.75	1.75	2.04	1.86	1.52
1998	5.43	2.22	6.57	2.62	4.23	11.4	5.1	2.7	2.6	6.5	1.70	1.72	2.05	1.81	1.50
1999	4.44	2.95	4.00	2.01	4.53	10.3	5.2	1.9	3.2	5.6	1.73	1.73	1.99	1.85	1.50
2000	5.63	3.75	4.68	3.21	4.74	9.8	4.5	2.3	3.7	4.7	1.73	1.77	2.08	1.85	1.54
2001	2.58	0.82	3.83	2.09	1.56	9.2	4.2	2.3	3.3	4.0	1.73	1.74	1.95	1.78	1.57
2002	1.68	0.47	0.31	1.44	2.07	9.1	4.3	3.2	3.9	4.0	1.72	1.72	1.93	1.75	1.65
2003	1.99	0.39	2.44	0.92	2.39	9.1	5.5	3.4	4.6	4.9	1.76	1.76	1.99	1.80	1.71
2004	3.93	2.67	8.10	3.96	4.32	8.9	5.3	3.2	4.5	5.5	1.80	1.78	2.04	1.83	1.75
2005	2.78	2.34	6.70	2.62	2.82	8.5	4.9	2.7	4.7	7.8	1.80	1.80	2.05	1.84	1.77
2006	4.06	3.91	5.00	2.40	4.69	7.8	4.0	2.9	3.5	7.1	1.84	1.85	2.08	1.90	1.85
2007	5.18	0.91	9.35	2.93	3.40	6.9	3.8	2.3	2.6	6.2	1.83	1.84	2.09	1.90	1.88
2008	0.72	− 0.51	1.52	0.38	− 0.56	6.4	3.5	3.0	2.6	6.2	1.85	1.89	2.15	1.96	1.91
2009	− 8.27	− 4.91	− 6.94	− 1.62	− 5.18	8.4	6.1	7.4	3.2	8.4	1.86	1.84	2.23	1.98	1.94
2010	2.99	1.87	− 3.56	0.60	5.99	8.5	7.6	7.7	3.7	8.7	1.87	1.87	2.20	1.95	1.98
2011	2.57	1.34	1.99	0.97	2.66	7.9	7.7	7.2	3.3	7.9	1.83	1.75	2.02	1.88	1.90
2012	− 1.43	0.23	1.22	2.75	− 0.29	7.8	7.7	6.2	3.3	8.1	1.80	1.73	2.04	1.85	1.91
2013	− 0.76	0.93	4.41	1.00	1.24	8.3	7.2	5.5	3.6	8.2	1.75	1.67	1.93	1.78	1.89
2014	− 0.63	1.68	1.93	1.92	2.60	8.8	6.8	5.1	3.6	8.1	1.71	1.69	1.93	1.75	1.88
2015	0.56	1.61	4.47	1.97	4.42	9.4	6.3	4.0	4.3	7.4	1.65	1.71	1.80	1.72	1.85
2016	2.63	3.25	7.35	1.07	2.41	8.8	6.0	3.0	4.7	7.0	1.57	1.79	1.75	1.71	1.85
2017	3.05	2.04	4.60	2.32	2.41	8.6	5.8	2.7	4.2	6.7	1.49	1.75	1.71	1.62	1.78
2018											1.40	1.73	1.71	1.56	1.76
2019											1.35	1.70	1.75	1.53	1.71

*Source*: Elaboration by the authors based on World Bank data for GDP and Eurostat for TFR and unemployment rate 1980–2015. Unemployment rates 1970–79 from Furaker et al. (1990)

**Table 3:** Relative risk of births by parity, 1987–1999 and 2003–2017

	Relative risk of first birth					Relative risk of second birth					Relative risk of third birth					Relative risk of fourth birth				
	FI	DK	NO	SW	IS	FI	DK	NO	SW	IS	FI	DK	NO	SW	IS	FI	DK	NO	SW	IS
1987	0.95	0.98	0.98	0.88	0.94	0.87	0.89	0.94	0.87	0.91	0.76	0.80	0.80	0.83	0.79	0.80	0.79	0.87	0.81	0.84
1988	0.98	0.98	1.01	0.92	1.03	0.94	0.96	0.97	0.94	1.00	0.87	0.85	0.87	0.90	0.93	0.86	0.81	0.93	0.87	0.88
1989	0.98	1.00	1.02	0.96	0.96	0.97	0.99	0.99	0.95	0.95	0.91	0.92	0.95	0.94	0.93	0.92	0.96	0.98	0.91	0.91
1990	1.00	1.00	1.00	1.00	1.00	1.00	1.00	1.00	1.00	1.00	1.00	1.00	1.00	1.00	1.00	1.00	1.00	1.00	1.00	1.00
1991	1.01	0.98	0.98	0.96	0.95	1.00	1.01	0.95	0.98	0.91	0.99	0.99	1.01	1.00	0.94	0.98	0.94	1.07	0.96	1.00
1992	1.01	0.98	0.93	0.89	0.92	1.03	1.08	0.94	0.96	0.93	1.04	1.12	0.99	0.94	0.91	1.04	1.04	1.04	0.95	0.99
1993	0.99	0.95	0.88	0.83	0.90	1.02	1.07	0.92	0.90	0.99	1.00	1.06	0.98	0.85	0.93	0.98	1.03	1.06	0.82	0.96
1994	0.97	0.97	0.87	0.79	0.88	1.04	1.11	0.94	0.89	0.92	1.04	1.17	0.99	0.77	0.93	1.00	1.09	1.07	0.76	0.85
1995	0.94	0.96	0.85	0.73	0.87	1.03	1.11	0.96	0.82	0.84	1.04	1.19	1.01	0.68	0.93	1.03	1.08	0.99	0.64	0.79
1996	0.91	0.93	0.85	0.68	0.82	1.00	1.06	0.99	0.77	0.96	1.00	1.10	1.05	0.58	0.90	0.98	1.01	1.03	0.56	0.89
1997	0.91	0.88	0.81	0.64	0.84	0.99	1.09	0.97	0.75	0.86	0.96	1.10	1.03	0.54	0.80	0.97	0.99	1.03	0.55	0.79
1998	0.87	0.86	0.79	0.62	0.84	0.97	1.09	0.96	0.76	0.95	0.95	1.06	1.00	0.57	0.77	0.95	0.95	0.98	0.57	0.81
1999	0.89	0.86	0.79	0.62	0.78	0.98	1.10	0.97	0.76	0.90	1.01	1.11	1.03	0.59	0.80	1.02	0.99	1.06	0.62	0.74
2003	1.00	0.91	0.93	0.93	1.04	0.96	0.89	0.89	0.97	0.87	0.93	0.84	0.91	0.91	0.80	0.86	0.86	0.86	0.91	0.78
2004	1.01	0.93	0.94	0.93	0.96	0.97	0.92	0.91	0.99	0.94	0.96	0.87	0.91	0.93	0.87	0.99	0.90	0.88	0.92	0.98
2005	1.00	0.93	0.94	0.93	0.99	0.97	0.95	0.93	1.00	0.93	0.96	0.90	0.92	0.92	0.90	0.94	0.92	0.89	0.93	0.85
2006	1.01	0.97	0.97	0.97	1.01	0.99	0.99	0.99	1.02	0.97	0.98	0.94	0.95	1.00	0.86	0.97	0.96	0.93	0.99	0.82
2007	1.00	0.96	0.95	0.98	0.98	0.99	0.99	0.98	1.02	0.99	0.98	0.96	0.95	1.01	0.92	0.96	0.97	0.97	0.98	1.02
2008	1.00	1.00	1.00	1.00	1.00	1.00	1.00	1.00	1.00	1.00	1.00	1.00	1.00	1.00	1.00	1.00	1.00	1.00	1.00	1.00
2009	1.01	0.96	0.99	1.02	0.98	1.02	0.97	1.03	1.02	1.07	1.01	0.95	1.02	1.01	1.06	0.99	0.92	0.97	1.01	1.07
2010	1.01	0.97	0.95	1.02	0.93	1.02	0.99	1.01	1.04	1.04	1.00	0.94	0.99	1.05	1.19	0.97	0.91	0.93	1.04	1.20
2011	0.96	0.89	0.89	0.96	0.81	1.01	0.92	0.97	1.00	1.00	0.99	0.84	0.97	0.99	1.03	1.02	0.83	0.90	1.00	0.95
2012	0.95	0.86	0.85	0.96	0.86	0.99	0.90	0.98	0.99	0.97	0.98	0.84	0.95	1.00	1.03	0.97	0.82	0.88	1.04	1.02
2013	0.90	0.83	0.82	0.92	0.79	0.99	0.87	0.96	0.99	0.93	0.97	0.80	0.90	1.01	0.96	0.99	0.80	0.81	1.01	0.89
2014	0.88	0.82	0.79	0.90		0.98	0.91	0.96	1.01		0.94	0.80	0.89	1.02		0.93	0.81	0.81	1.05	
2015	0.85	0.83	0.76	0.89		0.96	0.94	0.96	1.01		0.88	0.85	0.89	0.98		0.86	0.82	0.81	0.98	
2016	0.80	0.88	0.73	0.88		0.91	0.99	0.98	1.01		0.83	0.91	0.89	1.01		0.87	0.89	0.85	1.01	
2017	0.73	0.86	0.67	0.85		0.87	0.99	0.94	0.98		0.84	0.92	0.82	0.99		0.84	0.85	0.79	1.01	

*Source*: Elaboration of the authors based on each country’s register data. Baseline years are 1990 and 2008, respectively

**Table 4:** Relative risk of births by parity, 1987–1999 and 2003–2017. Women aged 16–29

	Relative risk of first birth					Relative risk of second birth					Relative risk of third birth					Relative risk of fourth birth				
	FI	DK	NO	SW	IS	FI	DK	NO	SW	IS	FI	DK	NO	SW	IS	FI	DK	NO	SW	IS
1987	0.96	1.00	0.99	0.89		0.86	0.89	0.95	0.86		0.73	0.81	0.81	0.83		0.76	0.76	0.89	0.74	
1988	0.99	0.99	1.02	0.93		0.95	0.96	0.98	0.93		0.85	0.86	0.90	0.89		0.88	0.80	1.05	0.88	
1989	0.98	1.00	1.02	0.97	0.96	0.98	0.98	0.98	0.95	0.96	0.93	0.90	0.98	0.94	0.95	1.02	0.92	1.14	0.86	1.04
1990	1.00	1.00	1.00	1.00	1.00	1.00	1.00	1.00	1.00	1.00	1.00	1.00	1.00	1.00	1.00	1.00	1.00	1.00	1.00	1.00
1991	1.01	0.96	0.97	0.95	0.95	1.00	1.01	0.93	0.98	0.91	1.03	1.01	1.00	0.98	0.89	1.02	0.93	1.06	0.93	0.90
1992	1.01	0.95	0.91	0.86	0.91	1.04	1.06	0.91	0.94	0.92	1.04	1.11	0.97	0.91	0.85	1.15	1.13	1.04	0.96	1.05
1993	0.96	0.91	0.86	0.79	0.87	1.03	1.06	0.89	0.88	1.00	1.05	1.02	0.96	0.82	0.89	1.06	0.91	1.10	0.77	0.89
1994	0.94	0.92	0.83	0.73	0.84	1.03	1.08	0.92	0.87	0.93	1.07	1.14	0.93	0.73	0.91	1.17	1.00	1.05	0.71	0.90
1995	0.89	0.90	0.81	0.66	0.84	1.00	1.06	0.92	0.79	0.78	1.08	1.17	0.93	0.61	0.92	1.15	1.08	1.00	0.58	0.81
1996	0.85	0.86	0.80	0.60	0.79	0.98	1.02	0.94	0.72	0.91	1.03	1.09	0.99	0.51	0.84	1.12	0.99	1.10	0.48	1.13
1997	0.84	0.80	0.75	0.56	0.80	0.97	1.05	0.92	0.71	0.83	0.98	1.08	0.97	0.47	0.81	1.13	0.99	0.97	0.51	0.69
1998	0.80	0.77	0.72	0.52	0.79	0.95	1.02	0.90	0.69	0.95	0.95	1.06	0.95	0.48	0.79	1.13	0.87	1.06	0.53	0.80
1999	0.82	0.75	0.71	0.51	0.74	0.94	1.04	0.91	0.70	0.85	1.00	0.99	0.95	0.51	0.80	1.20	1.01	1.04	0.61	0.81
2003	1.04	0.93	0.95	0.96		0.98	0.91	0.88	0.97		0.91	0.81	0.86	0.93		0.87	0.68	0.74	0.93	
2004	1.04	0.94	0.95	0.93		0.99	0.93	0.88	0.98		0.94	0.87	0.87	0.91		1.02	1.00	0.86	1.07	
2005	1.03	0.93	0.93	0.92		0.97	0.98	0.90	0.98		0.95	0.89	0.95	0.93		1.09	1.06	0.90	1.01	
2006	1.03	0.98	0.99	0.95		0.99	1.00	0.96	1.00		0.95	0.99	0.94	1.01		1.01	0.91	0.85	1.08	
2007	1.00	0.97	0.95	0.96	0.97	0.99	1.01	0.98	0.99	1.02	0.98	0.93	0.95	0.98	0.81	1.01	0.95	0.92	0.97	3.78
2008	1.00	1.00	1.00	1.00	1.00	1.00	1.00	1.00	1.00	1.00	1.00	1.00	1.00	1.00	1.00	1.00	1.00	1.00	1.00	1.00
2009	1.02	0.95	0.99	1.01	0.99	1.02	0.96	1.03	1.01	1.07	1.02	0.94	0.99	1.00	0.96	1.09	0.98	0.93	1.15	2.86
2010	1.00	0.95	0.94	1.00	0.92	1.03	0.94	0.98	1.03	1.03	1.01	0.92	0.96	1.08	0.91	1.03	1.01	0.86	1.12	4.84
2011	0.93	0.87	0.87	0.96	0.80	1.03	0.89	0.94	0.99	1.06	1.01	0.75	0.92	0.98	0.95	1.03	0.78	0.78	1.08	1.63
2012	0.93	0.84	0.82	0.94	0.84	1.00	0.88	0.97	0.98	0.99	0.98	0.77	0.93	1.00	0.78	0.96	0.77	0.79	1.10	1.10
2013	0.86	0.79	0.77	0.89	0.77	0.98	0.82	0.93	0.98	0.90	0.98	0.70	0.88	1.04	0.73	1.00	1.01	0.79	1.07	2.29
2014	0.83	0.78	0.73	0.88		0.98	0.88	0.93	1.05		1.01	0.72	0.84	1.06		0.97	0.85	0.70	1.20	
2015	0.79	0.79	0.70	0.87		0.97	0.93	0.94	1.06		0.85	0.81	0.85	1.02		0.94	0.87	0.87	1.12	
2016	0.72	0.83	0.65	0.87		0.90	0.98	0.95	1.07		0.85	0.85	0.90	1.04		0.91	0.81	0.85	1.11	
2017	0.65	0.81	0.58	0.83		0.87	1.00	0.92	1.05		0.82	0.87	0.82	1.01		0.85	0.85	0.87	1.06	

*Source*: Elaboration of the authors based on each country’s register data. Baseline years are 1990 and 2008, respectively

**Table 5:** Relative risk of births by parity, 1987–1999 and 2003–2017. Women aged 30–45

	Relative risk of first birth					Relative risk of second birth					Relative risk of third birth					Relative risk of fourth birth				
	FI	DK	NO	SW	IS	FI	DK	NO	SW	IS	FI	DK	NO	SW	IS	FI	DK	NO	SW	IS
1987	0.91	0.89	0.90	0.85		0.88	0.88	0.92	0.89		0.78	0.80	0.78	0.83		0.81	0.80	0.86	0.83	
1988	0.94	0.92	0.96	0.89		0.94	0.95	0.95	0.95		0.88	0.84	0.84	0.90		0.86	0.81	0.90	0.87	
1989	0.96	0.98	0.98	0.94	0.90	0.96	0.98	0.99	0.96	0.92	0.90	0.92	0.94	0.94	0.92	0.89	0.97	0.95	0.92	0.88
1990	1.00	1.00	1.00	1.00	1.00	1.00	1.00	1.00	1.00	1.00	1.00	1.00	1.00	1.00	1.00	1.00	1.00	1.00	1.00	1.00
1991	1.03	1.05	1.05	1.01	0.94	1.00	1.02	0.99	0.99	0.91	0.97	0.99	1.01	1.01	0.97	0.98	0.94	1.07	0.97	1.02
1992	1.05	1.11	1.04	1.01	1.05	1.02	1.11	0.99	1.00	0.97	1.04	1.12	1.00	0.95	0.94	1.01	1.02	1.04	0.95	0.98
1993	1.09	1.12	1.02	1.02	1.18	1.01	1.10	0.98	0.94	1.00	0.98	1.08	1.00	0.87	0.95	0.96	1.06	1.05	0.83	0.96
1994	1.07	1.20	1.09	1.02	1.30	1.06	1.16	0.97	0.93	0.92	1.03	1.18	1.02	0.79	0.94	0.96	1.11	1.07	0.77	0.84
1995	1.10	1.25	1.09	0.98	1.26	1.06	1.17	1.01	0.88	0.97	1.03	1.21	1.06	0.72	0.94	1.00	1.08	0.99	0.66	0.79
1996	1.11	1.25	1.13	0.96	1.17	1.03	1.12	1.07	0.85	1.08	0.99	1.12	1.08	0.62	0.93	0.95	1.01	1.02	0.58	0.86
1997	1.12	1.25	1.13	0.95	1.29	1.03	1.16	1.04	0.82	0.92	0.96	1.12	1.06	0.57	0.80	0.93	0.99	1.03	0.55	0.80
1998	1.09	1.24	1.14	0.98	1.32	1.00	1.18	1.05	0.85	0.97	0.95	1.06	1.03	0.60	0.78	0.91	0.96	0.97	0.58	0.81
1999	1.10	1.32	1.18	1.02	1.22	1.02	1.20	1.07	0.86	1.00	1.01	1.15	1.07	0.62	0.80	0.98	0.99	1.05	0.62	0.73
2003	0.92	0.88	0.90	0.88		0.94	0.88	0.91	0.97		0.95	0.84	0.92	0.90		0.86	0.88	0.87	0.91	
2004	0.95	0.91	0.92	0.91		0.96	0.92	0.94	1.00		0.97	0.87	0.93	0.93		0.98	0.88	0.88	0.90	
2005	0.96	0.92	0.94	0.93		0.98	0.94	0.95	1.01		0.96	0.91	0.92	0.92		0.90	0.91	0.89	0.93	
2006	0.98	0.96	0.94	0.99		1.00	0.99	1.00	1.03		0.99	0.93	0.95	1.00		0.96	0.97	0.94	0.98	
2007	0.98	0.95	0.94	0.98	1.01	0.98	0.98	0.99	1.03	0.96	0.98	0.97	0.96	1.02	0.97	0.95	0.98	0.97	0.98	0.90
2008	1.00	1.00	1.00	1.00	1.00	1.00	1.00	1.00	1.00	1.00	1.00	1.00	1.00	1.00	1.00	1.00	1.00	1.00	1.00	1.00
2009	1.00	0.99	0.98	1.04	0.93	1.01	0.98	1.04	1.02	1.07	1.00	0.95	1.02	1.01	1.10	0.96	0.92	0.97	1.00	0.99
2010	1.02	1.00	0.99	1.05	0.97	1.01	1.01	1.03	1.05	1.05	1.00	0.94	1.00	1.04	1.28	0.95	0.90	0.94	1.03	1.06
2011	1.00	0.95	0.94	0.99	0.84	1.00	0.94	0.99	1.00	0.94	0.99	0.85	0.98	0.99	1.07	1.01	0.83	0.91	0.99	0.92
2012	0.98	0.92	0.94	1.00	0.93	0.98	0.92	0.99	1.00	0.95	0.98	0.86	0.95	1.00	1.12	0.98	0.83	0.89	1.04	1.01
2013	0.96	0.92	0.92	0.99	0.88	0.98	0.90	0.98	1.00	0.95	0.97	0.82	0.91	1.00	1.04	0.99	0.78	0.81	1.00	0.83
2014	0.94	0.92	0.92	0.97		0.97	0.93	0.98	1.00		0.92	0.82	0.90	1.01		0.92	0.81	0.82	1.03	
2015	0.93	0.96	0.91	0.93		0.96	0.96	0.98	0.99		0.89	0.86	0.90	0.97		0.84	0.82	0.80	0.96	
2016	0.91	1.01	0.91	0.92		0.92	0.99	1.00	0.99		0.83	0.92	0.89	1.01		0.86	0.90	0.85	1.00	
2017	0.85	1.00	0.86	0.87		0.87	0.98	0.95	0.96		0.84	0.93	0.82	0.99		0.83	0.85	0.78	1.00	

*Source*: Elaboration of the authors based on each country’s register data. Baseline years are 1990 and 2008, respectively

**Table 6:** Relative risk of births by parity and educational level, 1987–1999

		First birth						Second birth						Third birth						Fourth birth				
		Primary		Secondary		Tertiary		Primary		Secondary		Tertiary		Primary		Secondary		Tertiary		Primary		Secondary		Tertiary
*Finland*																								
1987	0.92		0.95		0.94		0.91		0.87		0.85		0.80		0.76		0.75		0.82		0.80		0.80	
1988	0.92		1.00		0.98		0.94		0.96		0.93		0.86		0.88		0.90		0.86		0.86		0.91	
1989	0.92		0.99		1.00		0.96		0.97		0.98		0.93		0.91		0.91		0.91		0.94		0.90	
1990	1.00		1.00		1.00		1.00		1.00		1.00		1.00		1.00		1.00		1.00		1.00		1.00	
1991	0.99		1.02		1.03		1.00		1.00		0.99		0.96		1.01		0.97		0.96		1.01		0.95	
1992	1.03		1.00		1.03		1.01		1.04		1.03		1.00		1.06		1.00		1.02		1.04		1.05	
1993	0.97		0.96		1.06		0.99		1.02		1.01		1.01		1.02		0.93		0.96		1.00		0.95	
1994	0.95		0.94		1.04		1.02		1.04		1.04		1.03		1.06		0.97		0.99		1.01		0.97	
1995	0.95		0.88		1.04		1.02		1.02		1.02		1.03		1.07		0.96		1.10		1.06		0.88	
1996	0.95		0.85		1.00		1.00		1.00		0.98		1.03		1.03		0.88		0.99		1.00		0.89	
1997	0.92		0.86		1.00		1.04		0.97		0.97		1.02		0.99		0.84		1.03		0.94		0.92	
1998	0.89		0.82		0.95		0.99		0.97		0.93		0.99		0.97		0.84		0.97		0.96		0.88	
1999	0.94		0.83		0.95		1.00		0.97		0.94		1.06		1.03		0.88		0.99		1.08		0.90	
*Denmark*																								
1987	1.02		0.93		0.92		0.93		0.87		0.86		0.81		0.79		0.87		0.85		0.68		0.95	
1988	1.01		0.93		0.96		0.97		0.95		0.96		0.85		0.86		0.85		0.86		0.70		0.87	
1989	1.02		0.98		0.98		1.00		0.98		0.97		0.92		0.91		0.92		1.00		0.89		0.78	
1990	1.00		1.00		1.00		1.00		1.00		1.00		1.00		1.00		1.00		1.00		1.00		1.00	
1991	0.96		0.99		1.01		1.01		1.04		0.98		0.96		1.06		0.97		0.95		0.91		0.84	
1992	1.00		0.98		0.99		1.06		1.11		1.05		1.08		1.14		1.15		1.09		0.91		0.95	
1993	0.96		0.96		0.97		1.05		1.11		1.04		1.02		1.12		1.03		1.07		0.91		0.97	
1994	0.97		1.00		1.01		1.05		1.15		1.11		1.11		1.25		1.10		1.16		0.95		1.00	
1995	0.99		0.97		1.01		1.08		1.17		1.03		1.15		1.28		1.08		1.15		1.00		0.80	
1996	0.95		0.95		0.98		1.00		1.10		1.06		1.07		1.17		1.02		1.11		0.89		0.77	
1997	0.89		0.90		0.97		1.03		1.12		1.09		1.07		1.15		1.04		1.10		0.88		0.76	
1998	0.86		0.87		0.95		0.99		1.13		1.10		1.01		1.11		1.00		1.02		0.85		0.78	
1999	0.85		0.86		0.98		0.96		1.15		1.11		1.01		1.18		1.08		1.06		0.89		0.82	
*Norway*																								
1987	1.03		0.92		0.98		0.98		0.92		0.93		0.79		0.80		0.84		0.85		0.89		1.01	
1988	1.04		0.97		1.02		1.01		0.95		0.95		0.88		0.86		0.88		0.90		0.95		1.01	
1989	1.03		0.99		1.02		1.00		0.98		0.99		0.95		0.95		0.98		1.02		0.97		0.95	
1990	1.00		1.00		1.00		1.00		1.00		1.00		1.00		1.00		1.00		1.00		1.00		1.00	
1991	0.97		0.99		1.01		0.95		0.95		0.95		1.01		1.01		1.00		1.04		1.08		1.04	
1992	0.89		0.97		0.95		0.92		0.94		0.93		0.93		0.99		1.01		1.00		1.04		1.06	
1993	0.83		0.94		0.90		0.90		0.93		0.92		0.91		1.01		0.99		1.03		1.04		1.06	
1994	0.79		0.95		0.90		0.92		0.94		0.92		0.94		0.98		1.01		0.98		1.12		0.98	
1995	0.78		0.93		0.91		0.92		0.97		0.94		0.98		1.00		1.02		0.92		0.99		0.97	
1996	0.76		0.94		0.92		0.96		0.99		0.97		0.97		1.06		1.04		1.02		1.02		0.92	
1997	0.71		0.91		0.88		0.93		0.97		0.94		0.96		1.04		1.01		1.01		1.03		0.89	
1998	0.69		0.88		0.88		0.90		0.96		0.96		0.91		1.03		0.96		0.94		0.98		0.88	
1999	0.68		0.85		0.95		0.90		0.98		0.95		0.96		1.02		0.99		1.04		1.06		0.89	
*Sweden*																								
1990	1.00		1.00		1.00		1.00		1.00		1.00		1.00		1.00		1.00		1.00		1.00		1.00	
1991	0.98		0.96		1.01		0.99		0.99		0.96		1.01		1.00		0.98		0.93		0.99		0.97	
1992	0.93		0.90		0.99		0.94		0.95		0.99		0.93		0.94		0.91		0.87		0.97		0.97	
1993	0.82		0.86		0.97		0.87		0.91		0.93		0.91		0.85		0.81		0.88		0.83		0.76	
1994	0.82		0.85		0.95		0.90		0.90		0.91		0.81		0.77		0.75		0.78		0.76		0.77	
1995	0.80		0.79		0.87		0.83		0.83		0.88		0.78		0.67		0.66		0.73		0.63		0.64	
1996	0.74		0.76		0.84		0.80		0.79		0.82		0.69		0.56		0.59		0.63		0.58		0.53	
1997	0.72		0.74		0.79		0.80		0.80		0.83		0.67		0.55		0.55		0.66		0.59		0.49	
1998	0.69		0.71		0.79		0.83		0.81		0.83		0.70		0.59		0.58		0.74		0.60		0.56	
1999	0.71		0.71		0.77		0.80		0.81		0.85		0.75		0.58		0.59		0.75		0.67		0.55	

*Source*: Elaboration of the authors based on each country’s register data. Baseline category is year 2008 for (upper) secondary-educated women

**Table 7:** Relative risk of births by parity and educational level, 2003–last year available

		First Birth						Second Birth						Third Birth						Fourth Birth				
		Primary		Secondary		Tertiary		Primary		Secondary		Tertiary		Primary		Secondary		Tertiary		Primary		Secondary		Tertiary
*Finland*																								
2003	1.05		0.99		0.98		0.96		0.99		0.96		0.94		0.91		0.97		0.90		0.83		0.88	
2004	1.08		1.00		0.99		1.01		0.99		0.97		0.98		0.93		0.99		0.92		0.98		1.06	
2005	1.06		0.99		1.01		0.95		0.98		0.99		0.94		0.95		0.98		0.90		0.93		1.00	
2006	1.08		0.99		1.02		0.96		1.01		1.00		0.96		0.98		1.00		0.95		0.94		1.05	
2007	1.06		0.98		1.01		0.98		0.99		0.99		0.99		0.95		1.01		0.95		0.99		0.94	
2008	1.00		1.00		1.00		1.00		1.00		1.00		1.00		1.00		1.00		1.00		1.00		1.00	
2009	1.05		1.01		1.00		0.94		1.02		1.03		1.02		0.98		1.03		1.02		0.97		0.99	
2010	1.02		0.99		1.02		0.97		1.04		1.01		1.02		1.00		1.00		0.94		1.00		0.94	
2011	0.92		0.96		0.97		0.94		1.02		1.01		1.01		0.98		1.00		1.04		1.04		0.97	
2012	0.95		0.95		0.95		0.93		1.00		0.99		1.00		0.99		0.95		0.98		0.99		0.95	
2013	0.88		0.91		0.90		0.85		0.99		1.00		1.01		1.00		0.93		0.99		1.02		0.95	
2014	0.86		0.87		0.90		0.84		0.98		0.99		0.97		0.96		0.91		0.94		0.99		0.84	
2015	0.77		0.86		0.86		0.83		0.97		0.97		0.98		0.89		0.84		0.90		0.93		0.76	
2016	0.70		0.81		0.82		0.82		0.91		0.93		0.89		0.84		0.80		0.96		0.92		0.77	
2017	0.60		0.74		0.76		0.79		0.88		0.87		0.81		0.86		0.81		0.89		0.85		0.81	
*Denmark*																								
2003	0.83		0.98		0.89		0.89		0.93		0.90		0.85		0.85		0.90		0.81		0.84		0.97	
2004	0.86		0.97		0.94		0.94		0.96		0.93		0.88		0.87		0.94		0.94		0.87		0.88	
2005	0.87		0.96		0.94		0.97		0.98		0.95		0.94		0.90		0.94		1.00		0.90		0.87	
2006	0.93		0.99		0.97		0.96		1.00		1.01		0.93		0.96		0.95		0.97		0.95		0.97	
2007	0.95		0.97		0.96		0.97		1.01		0.99		0.96		0.97		0.98		0.96		1.02		0.93	
2008	1.00		1.00		1.00		1.00		1.00		1.00		1.00		1.00		1.00		1.00		1.00		1.00	
2009	0.95		0.95		0.98		0.95		0.98		0.97		0.94		0.91		0.97		0.97		0.92		0.89	
2010	0.95		0.97		0.98		0.90		0.99		0.99		0.93		0.91		0.94		1.05		0.93		0.77	
2011	0.88		0.89		0.90		0.84		0.93		0.93		0.78		0.83		0.83		0.89		0.88		0.72	
2012	0.85		0.86		0.87		0.82		0.90		0.91		0.83		0.81		0.82		0.83		0.93		0.72	
2013	0.80		0.85		0.84		0.79		0.86		0.88		0.81		0.79		0.75		0.97		0.85		0.65	
2014	0.76		0.84		0.84		0.82		0.91		0.91		0.81		0.78		0.75		0.92		0.90		0.66	
2015	0.73		0.86		0.87		0.84		0.93		0.95		0.84		0.80		0.83		0.96		0.89		0.68	
2016	0.72		0.90		0.93		0.89		0.96		0.99		0.87		0.89		0.85		0.97		0.99		0.76	
2017	0.68		0.87		0.92		0.89		1.00		0.95		0.95		0.91		0.84		0.99		0.87		0.73	
*Norway*																								
2003	0.86		0.93		0.96		0.86		0.93		0.92		0.88		0.92		1.00		0.90		0.79		0.93	
2004	0.85		0.95		0.97		0.89		0.92		0.94		0.86		0.92		0.99		0.94		0.82		0.92	
2005	0.86		0.93		0.97		0.90		0.94		0.96		0.87		0.92		0.99		0.96		0.83		0.93	
2006	0.95		0.95		0.99		0.95		0.99		1.01		0.91		0.94		1.00		0.95		0.92		0.94	
2007	0.93		0.94		0.96		0.97		0.97		1.00		0.94		0.95		0.97		0.95		1.00		0.95	
2008	1.00		1.00		1.00		1.00		1.00		1.00		1.00		1.00		1.00		1.00		1.00		1.00	
2009	0.99		0.95		1.02		1.05		1.03		1.03		1.00		0.99		1.03		1.09		0.93		0.93	
2010	0.95		0.92		0.98		0.96		0.98		1.03		0.99		0.93		1.01		1.07		0.89		0.90	
2011	0.88		0.85		0.94		0.91		0.95		0.99		0.94		0.89		0.99		0.91		0.89		0.89	
2012	0.80		0.83		0.91		0.94		0.96		0.99		0.95		0.89		0.94		0.94		0.92		0.81	
2013	0.77		0.78		0.87		0.90		0.93		0.97		0.93		0.87		0.88		0.82		0.80		0.80	
2014	0.69		0.74		0.86		0.91		0.95		0.96		0.90		0.85		0.86		0.82		0.82		0.78	
2015	0.67		0.73		0.83		0.89		0.93		0.97		0.90		0.86		0.86		0.88		0.80		0.77	
2016	0.59		0.71		0.80		0.89		0.96		0.99		0.89		0.86		0.85		0.97		0.87		0.77	
2017	0.51		0.62		0.76		0.85		0.91		0.95		0.83		0.83		0.76		0.97		0.82		0.69	
*Sweden*																								
2003	0.97		1.01		0.94		0.95		1.01		1.02		0.94		0.94		0.96		0.97		0.92		0.94	
2004	0.91		0.98		0.95		0.92		1.03		1.05		0.96		0.92		1.02		1.01		0.91		0.94	
2005	0.90		0.98		0.94		0.93		1.01		1.05		0.97		0.94		0.96		0.89		0.94		0.99	
2006	0.95		1.01		0.98		0.94		1.04		1.05		1.03		1.00		1.03		1.11		1.00		0.96	
2007	0.98		0.99		0.97		1.00		1.03		1.02		0.99		1.03		1.02		1.08		0.96		0.96	
2008	1.00		1.00		1.00		1.00		1.00		1.00		1.00		1.00		1.00		1.00		1.00		1.00	
2009	1.00		1.03		1.03		0.99		1.01		1.02		1.06		1.01		1.00		1.19		1.01		0.93	
2010	1.00		1.03		1.05		1.00		1.04		1.04		1.17		1.06		1.01		1.18		1.03		1.00	
2011	0.96		0.97		0.99		0.99		1.01		0.98		1.06		1.02		0.94		1.08		1.07		0.89	
2012	0.92		0.96		1.00		0.97		0.99		0.97		1.11		1.04		0.91		1.19		1.12		0.92	
2013	0.88		0.92		0.97		0.94		0.99		0.97		1.06		1.08		0.92		1.11		1.07		0.90	
2014	0.84		0.91		0.95		0.95		1.05		0.97		1.21		1.07		0.93		1.10		1.20		0.92	
2015	0.76		0.88		0.95		0.96		1.02		0.97		1.12		1.06		0.88		1.16		1.05		0.84	
2016	0.68		0.88		0.91		0.91		1.03		0.97		1.13		1.08		0.91		1.14		1.13		0.86	
2017	0.63		0.86		0.86		0.85		1.00		0.95		1.13		1.08		0.88		1.23		1.06		0.90	

*Source*: Elaboration of the authors based on each country’s register data. Baseline category is year 2008 for upper-secondary educated women

## Footnotes

1 Childrearing, though, also entails the indirect cost of forgone career opportunities due to the time devoted to childcare instead of paid work. Theoretically, career interruptions thus offer momentum for having children because the opportunity cost is thereby minimal.

2 For Iceland, the latest data available are for 2013.

## References

[CR1] Andersen J, Ólafsson S, Daly M, Kangas O, Palme J (2019). Denmark: The welfare state as a victim of neoliberal economic failure?. Welfare and the Great Recession: A Comparative Study.

[CR2] Andersen, P. (1997). Macroeconomic developments in the Nordic countries. In: Monetary policy in the Nordic countries: Experiences since 1992. BIS Policy Paper no. 2, 188–229. Basel: Bank for International Settlements.

[CR3] Andersen SH, Jensen B, Nielsen BW, Skaksen JR (2017). Hvad vi ved om udsatte unge, historik, omfang og årsager.

[CR4] Andersson G (1999). Childbearing trends in Sweden 1961–1997. European Journal of Population.

[CR5] Andersson G (2000). The impact of labor-force participation on childbearing behavior: pro-cyclical fertility in Sweden during the 1980s and the 1990s. European Journal of Population.

[CR6] Andersson G (2002). Fertility developments in Norway and Sweden since the early 1960s. Demographic Research.

[CR7] Andersson G (2004). Childbearing developments in Denmark, Norway, and Sweden from the 1970s to the 1990s: A comparison. Demographic Research Special Collection.

[CR8] Andersson G, Kolk M (2016). Trends in childbearing, marriage and divorce in Sweden: An update with data up to 2012. Finnish Yearbook of Population Research.

[CR9] Bachmann R, Bayer C (2013). “Wait-and-See” business cycles?. Journal of Monetary Economics.

[CR10] Becker, G. S. (1960). An economic analysis of fertility. In NBER (ed.): *Demographic and Economic Change in Developed Countries,* 209–240. Columbia University Press, New York.

[CR11] Bellido H, Marcén M (2019). Fertility and the business cycle: the European case.

[CR12] Bloom N (2009). The impact of uncertainty shocks. Econometrica.

[CR13] Caltabiano M, Comolli CL, Rosina A (2017). The effect of the Great Recession on permanent childlessness in Italy. Demographic Research.

[CR14] Centers for Disease Control and Prevention (CDC) (2019). Births: Final data for 2018. *National Vital Statistics Report*, 68(13) (by Martin, J., Hamilton, B., Osterman M. and Driscoll, A.) 32501202

[CR15] Comolli CL (2017). The fertility response to the Great Recession in Europe and the United States: Structural economic conditions and perceived economic uncertainty. Demographic Research.

[CR16] Comolli CL, Bernardi F (2015). The causal effect of the great recession on childlessness of white American women. IZA Journal of Labor Economics.

[CR17] Comolli CL (2018). Finnish fertility: pro- or counter-cyclical?. Research on Finnish Society.

[CR18] Comolli CL (2019). Couples’ transition to parenthood in Finland: a tale of two recessions. Stockholm Research Reports in Demography.

[CR19] Dølvik JE, Oldervoll J, Ólafsson S, Daly M, Kangas O, Palme J (2019). Norway: Averting crisis through coordination and Keynesian welfare policies. Welfare and the Great Recession: A Comparative Study.

[CR20] Dommermuth, L., and Lappegård, T. (2017). Nedgangen i fruktbarheten fra 2010. Betydningen av utdanning, økonomisk aktivitet og økonomiske ressurser for førstefødsler og tredjefødsler [The decline in fertility since 2010. The impact of economic activity, economic resources and education on first births and third births]. SSB Reports 2017/12.

[CR21] DØR (2007). Dansk Økonomi, forår 2007.

[CR22] Einarsson, B.G., Gunnlaugsson, K., Ólafsson, T.T., and Pétursson, T.G. (2015). The long history of financial boom-bust cycles in Iceland – Part I: Financial crises. Central Bank of Iceland *Working Paper* no. 68.

[CR23] Einarsson, B.G., Gunnlaugsson, K., Ólafsson, T.T., and Pétursson, T.G. (2016). The long history of financial boom-bust cycles in Iceland – Part II: Financial cycles. Central Bank of Iceland *Working Paper* no. 72.

[CR24] Elder G (1974). Children of the Great Depression.

[CR25] Erhvervs- og Vækstministeriet (2013). Den finansielle krise i Danmark – årsager, konsekvenser og læring.

[CR26] Esping-Andersen G, Gallie D, Hemerijck A, Myles J (2002). Why We Need a New Welfare State.

[CR27] Eydal GB, Ólafsson S, Ostner I, Schmitt C (2008). Family policy in Iceland: An Overview. Family Policies in the Context of Family Change. The Nordic Countries in Comparative Perspective.

[CR28] Fishback P, Haines M, Kantor S (2007). Births, deaths, and the New Deal relief during the Great Depression. The Review of Economics and Statistics.

[CR29] Furaker B, Johansson L, Lind J (1990). Unemployment and labour market policies in the Scandinavian countries. Acta Sociologica.

[CR30] Goldstein J, Kreyenfeld M, Jasilioniene A, Örsal DDK (2013). Fertility reactions to the "Great Recession" in Europe: Recent evidence from order-specific data. Demographic Research.

[CR31] Haataja A (2005). Outcomes of the two 1990s family policy reforms at the turn of the 2000s in Finland. Yearbook of the Population Research in Finland.

[CR32] Hall PA, Soskice D (2001). Varieties of Capitalism: The Institutional Foundations of Comparative Advantage.

[CR33] Heikkilä, M., and Uusitalo, H. (1997). *Leikkausten hinta. Tutkimuksia sosiaaliturvan leikkauksista ja niiden vaikutuksista 1990-luvun Suomessa [The Price of the Cuts]* (No. 208). Report.

[CR34] Hiilamo H (2017). Fertility response to economic recessions in Finland 1991–2015. Finnish Yearbook of Population Research.

[CR200] Hiilamo H (2019). Why fertility has been declining in Finland after the Global Recession?. Finnish Yearbook of Population Research.

[CR35] Hiilamo H, Kangas O (2009). Trap for women or freedom to choose? The struggle over cash for child care schemes in Finland and Sweden. Journal of Social Policy.

[CR36] Hoem, B. (2000). Entry into motherhood in Sweden: The influence of economic factors on the rise and fall in fertility, 1986–1997. *Demographic Research,* 2(4).

[CR37] Hoem B, Hoem JM (1996). Sweden’s family policies and roller-coaster fertility. Jinko Mondai Kenkyu (Journal of Population Problems).

[CR38] Hoem JM (1991). La standardisation indirecte améliorée et son application à la divortialité en Suède (1971–1989). Population.

[CR39] Hoem JM (1993). Classical demographic methods of analysis and modern event-history techniques. IUSSP: 22nd International Population Conference Montreal, Canada.

[CR40] Huttunen K, Kellokumpu J (2016). The effect of job displacement on couples’ fertility decisions. Journal of Labor Economics.

[CR41] Jalovaara M, Fasang A (2017). From never partnered to serial cohabitors: union trajectories to childlessness. Demographic Research.

[CR42] Jensen TL, Johannesen N (2017). The consumption effects of the 2007–2008 financial crisis: Evidence from households in Denmark. American Economic Review.

[CR43] Jonassen AB, Skaksen JR (2019). Status på beskæftigelsen i Danmark. Hvordan udvikler beskæftigelsen sig i Danmark?.

[CR44] Jónsson AK (2017). Childbearing trends in Iceland, 1982–2013: Fertility timing, quantum, and gender preferences for children in a Nordic context. Demographic Research.

[CR45] Jónsson AK (2018). Family policies, childbearing, and economic crisis: The case of Iceland. Demographic Research.

[CR46] Jonung L, Kiander J, Vartia P (2009). The Great Financial Crisis in Finland and Sweden: The Nordic Experience of Financial Liberalization.

[CR47] Kangas O, Ólafsson S, Daly M, Kangas O, Palme J (2019). Finland: From the deep Crisis of the 1990s to the Great Recession. Welfare and the Great Recession: A Comparative Study.

[CR48] Kohler HP, Billari FC, Ortega JA (2002). The emergence of lowest-low fertility in Europe during the 1990s. Population and Development Review.

[CR49] Kravdal Ø (2002). The impact of individual and aggregate unemployment on fertility in Norway. Demographic Research.

[CR50] Kumlin, S., Stadelmann-Steffen, I., and Haugsgjerd, A. (2018). Trust and the welfare state. In: The Oxford Handbook of Social and Political Trust, 384–408. Oxford Handbooks Online.

[CR51] Lanzieri, G. (2013). Towards a “baby recession” in Europe? Differential fertility trends during the economic crisis. Statistics in Focus 13/2013.

[CR52] Lee R (2003). The demographic transition: three centuries of fundamental change. Journal of Economic Perspectives.

[CR53] Lesthaeghe R (2010). The unfolding story of the second demographic transition. Population and Development Review.

[CR54] Lin C-Y-Y, Edvinsson L, Chen J, Beding T (2014). National Intellectual Capital and the Financial Crisis in Denmark, Finland, Iceland, Norway, and Sweden.

[CR55] Madsen PK, Campbell JL, Hall JA, Pedersen OK (2006). How can it possibly fly? The paradox of a dynamic labour market in a Scandinavian welfare state. National Identity and the Varieties of Capitalism: The State of Denmark.

[CR56] Matysiak, A., Sobotka, T., and Vignoli, D. (2020). The *Great Recession* and fertility in Europe: A sub-national analysis. *European Journal of Population*, online first: 03 April 2020. 10.1007/s10680-020-09556-y PMC7864853 33597835

[CR57] Mau S, Mewes J, Schöneck NM (2012). What determines subjective socio-economic insecurity? Context and class in comparative perspective. Socio-Economic Review.

[CR58] McDonald P (2000). Gender equity, social institutions and the future of fertility. Journal of Population Research.

[CR59] Miettinen, A. and Jalovaara, M. (2019). Unemployment delays first birth but not for all. Life stage and educational differences in the effects of employment uncertainty on first births. *Advances in Life Course Research*. Online November. 10.1016/j.alcr.2019.100320 36726257

[CR60] Mills M, Blossfeld HP (2003). Globalization, uncertainty and changes in early life courses. Zeitschrift für Erziehungswissenschaft.

[CR61] Myrdal A (1945). Nation and Family.

[CR62] Nätti, J., Kinnunen, U., Happonen, M., Mauno, S., and Sallinen, M. (2001). Perceived job insecurity among Finnish employees in 1990–2000: Prevalence and antecedents. In: J. Kalela, J. Kiander, U. Kivikuru, H.A. Loikkanen and J. Simpura (eds.) *Down from the Heavens, Up from the Ashes*, 484–506. Government Institute for Economic Research. Helsinki.

[CR63] Neels K, Theunynck Z, Wood J (2013). Economic recession and first births in Europe: recession-induced postponement and recuperation of fertility in 14 European countries between 1970 and 2005. International Journal of Public Health.

[CR64] Neyer G, Andersson G (2008). Consequences of family policies on childbearing behavior: effects or artifacts?. Population and Development Review.

[CR65] Neyer, G., and Andersson, G., Hoem, J.M., Rønsen, M., and Vikat, A. (2006). In: H. Bertram, H. Krüger, C.K. Spieß (eds.) *Wem gehört die Familie der Zukunft?: Expertisen zum 7. Familienbericht der Bundesregierung*, 207–233. Opladen: Budrich.

[CR66] Ólafsson S (2003). Welfare trends of the 1990s in Iceland. Scandinavian Journal of Public Health.

[CR67] Ólafsson, S. (2011). Iceland’s financial crisis and level of living consequences. University of Iceland, *Social Research Centre Working Paper* 3:2011.

[CR68] Ólafsson, S. (2019). Iceland’s strategy of redistribution. In: Ólafsson, S., Daly, M., Kangas, O., and Palme, J. (eds.). *Welfare and the Great Recession: A Comparative Study*, 132–154. Oxford Scholarship Online.

[CR69] Ólafsson, S., Daly, M., Kangas, O., and Palme, J. (eds.). (2019). *Welfare and the Great Recession: A Comparative Study*. Oxford Scholarship Online.

[CR71] Oppenheimer VK (1994). Women's rising employment and the future of the family in industrial societies. Population and Development Review.

[CR70] Oppenheimer VK (1997). Women's employment and the gain to marriage: The specialization and trading model. Annual Review of Sociology.

[CR72] Örsal K, Goldstein J (2018). The changing relationship between unemployment and total fertility. Population Studies.

[CR73] Palme J, Ólafsson S, Daly M, Kangas O, Palme J (2019). Sweden: In times of two crises. Welfare and the Great Recession: A Comparative Study.

[CR74] Przeworski A, Teune H (1970). The Logic of Comparative Social Inquiry.

[CR75] Ranjan P (1999). Fertility behaviour under income uncertainty. European Journal of Population.

[CR76] Rasmussen S, Nätti J, Larsen TP, Ilsøe A, Garde AH (2019). Nonstandard Employment in the Nordics-Toward Precarious Work?. Nordic Journal of Working Life Studies.

[CR77] Rønsen M (2004). Fertility and public policies-Evidence from Norway and Finland. Demographic research.

[CR78] Schneider D (2015). The great recession, fertility, and uncertainty: Evidence from the United States. Journal of Marriage and Family.

[CR79] Seltzer N (2019). Beyond the Great Recession: labor market polarization and ongoing fertility decline in the United States. Demography.

[CR80] Sobotka T, Skirbekk V, Philipov D (2011). Economic recession and fertility in the developed world. Population and Development Review.

[CR81] Suni P, Vihriälä V (2016). Finland and Its Northern Peers in the Great Recession.

[CR82] Vignoli, D., Guetto, R., Bazzani, G., Pirani, E., and Minello, A. (2020). *Economic Uncertainty and Fertility in Europe: Narratives of the Future* (No. 2020_01). Università degli Studi di Firenze, Dipartimento di Statistica, Informatica, Applicazioni" G. Parenti".

[CR83] Vikat A (2004). Women’s labor force attachment and childbearing in Finland. Demographic Research Special Collection.

